# Whole-food diet therapies for children with Crohn’s disease: a systematic review

**DOI:** 10.1177/17562848251355436

**Published:** 2025-07-23

**Authors:** Cathy Guo, Julia Fox, Kristie Bell, Danielle Gallegos, Lynda J. Ross

**Affiliations:** Faculty of Exercise and Nutrition Sciences, Queensland University of Technology, 149 Victoria Park Road, Kelvin Grove, Brisbane, QLD 4059, Australia; Children’s Health Queensland Hospital and Health Service, South Brisbane, QLD, Australia; Children’s Health Queensland Hospital and Health Service, South Brisbane, QLD, Australia; Faculty of Exercise and Nutrition Sciences, Queensland University of Technology, Brisbane, QLD, Australia; Centre for Childhood Nutrition Research, South Brisbane, QLD, Australia; Faculty of Exercise and Nutrition Sciences, Queensland University of Technology, Brisbane, QLD, Australia

**Keywords:** diet, inflammatory bowel disease, mucosal healing, paediatric Crohn’s disease, remission, whole-food diet therapies

## Abstract

**Background:**

Children with Crohn’s disease (CD) experience gastrointestinal symptoms that impair nutrition, growth and quality of life. Exclusive enteral nutrition is recommended as a first-line remission induction treatment. However, compliance is challenging due to monotony and the social impact of excluding whole foods, increasing interest in whole-food diet therapies.

**Objectives:**

This systematic review aimed to summarise current evidence regarding the impact of whole-food therapies on clinical remission (as defined by each study using validated measures) and related health outcomes in children with CD.

**Design:**

We performed a systematic review of studies assessing whole-food interventions in children with CD.

**Data sources and methods:**

A systematic search was conducted in electronic databases for research published in English from 1 January 2012 to 16 August 2024. Randomised controlled trials (RCTs), quasi-experimental studies, cohort studies, case-control studies and case series were included.

**Results:**

Twenty-eight studies (*n* = 4 RCTs, *n* = 3 non-RCTs, *n* = 16 observational studies, *n* = 5 post hoc analyses) met inclusion criteria and examined six types of diets. Most of the children being treated had uncomplicated, mild–moderate disease activity and were on concomitant medications. Those on whole-food therapies demonstrated a median clinical remission rate of 75% (interquartile range 62%–85%; *n* = 18 studies), mucosal improvement and healing (*n* = 5/6 studies), improved inflammatory biomarkers (*n* = 18/19 studies) and enhanced growth parameters (*n* = 11/13 studies). Outcomes related to microbial changes were inconsistent. Overall, studies were low–medium quality due to small, non-randomised, uncontrolled studies using a variety of concomitant medications and different definitions for clinical remission, preventing definitive conclusions.

**Conclusion:**

The findings suggest whole-food diet therapies can potentially be used to treat children with mild to moderate CD and that a flexible, nutrient-balanced dietary approach tailored to the individual child may be possible. However, large-scale, RCTs with standardised outcome measures are needed to further support the routine use of whole-food therapies in paediatric CD.

**Trial registration:**

PROSPERO registration number CRD42024580134.

## Introduction

Crohn’s Disease (CD) is an inflammatory bowel disease (IBD) characterised by transmural inflammation and lesions presenting in various parts of the gastrointestinal tract.^1^ The pathogenesis of CD is a complex interplay of genetic predispositions, gut microbiome, the immune system and environmental factors.^2^ Dysbiosis, characterised by an imbalance in the gut microbial composition and function, is a central feature in the progression of CD.^2^ Metagenomic studies have demonstrated that individuals with CD exhibit reduced overall microbial diversity, an increase in potentially pro-inflammatory microbiota and a decrease in beneficial microorganisms.^3^ The dysbiosis can trigger an excessive immune response against the body’s own cells, leading to inflammation and damage to the mucosal barrier.^2,3^

While the pathogenesis of CD in adults and children may be comparable, paediatric-onset CD is often more aggressive, characterised by rapid progression and increased disease activity, resulting in symptoms, including abdominal pain, rectal bleeding and diarrhoea.^4^ Children with CD face additional challenges such as chronic malnutrition, sarcopenia, poor bone density and growth failure during critical developmental periods.^4,5^ Consequently, treatment must address multiple objectives: symptom relief, inflammation reduction, clinical remission, mucosal healing, improvement of growth and quality of life (QoL).^6^ Treatment efficacy is evaluated through clinical assessment of symptoms, growth parameters and validated indices like the Paediatric Crohn’s Disease Activity Index (PCDAI), alongside laboratory inflammatory biomarkers C-reactive protein (CRP), erythrocyte sedimentation rate (ESR), faecal calprotectin (FC). Endoscopic evaluation and imaging studies are also employed to assess objective signs of disease activity and mucosal healing.^4^

Extensive evidence has shown that exclusive enteral nutrition (EEN) is as effective as corticosteroids in inducing remission in children with CD.^7^ EEN is recommended as a first-line induction treatment for children with active, mild to moderate luminal CD due to fewer side effects, better support for growth and superior mucosal healing.^7–9^ However, the monotony, poor palatability and social challenges associated with EEN result in poor compliance and mental health impacts making it unsuitable for long-term use.^10^ Studies also consistently report inappropriate dietary habits among children and adolescents with IBD, marked by imbalanced intake of energy, macro and micronutrients.^11,12^ Common findings include insufficient intake of carbohydrates, fibre, calcium and vitamin A compared to recommended levels and healthy controls.^12^ Additionally, Italian children with IBD showed poor adherence to a Mediterranean diet (MD) pattern, highlighting potential suboptimal dietary quality in this population.^11^ Given diet is a modifiable risk factor for managing intestinal dysbiosis and supporting nutrition and growth,^13^ interest is rising in the use of whole-food diet therapies for treating paediatric CD.

While individual studies have investigated these whole-food diet therapies, to date, there has been no attempt to synthesise and evaluate their impact on disease management and health, specifically in children and adolescents with CD. This systematic literature review aims to (1) summarise the current evidence on the effectiveness of whole-food diets in achieving or maintaining clinical remission and (2) explore related outcomes, including any impacts on mucosal healing, inflammatory biomarkers, growth and nutritional parameters and gut microbiome and metabolites in children with CD.

## Materials and methods

This systematic review adhered to the Preferred Reporting Item for Systematic Reviews and Meta-Analyses (PRISMA) statement^14^ (Table S1) and was registered on the International Prospective Systems Review Registry (PROSPERO; #CRD42024580134).

### Search strategy

A comprehensive and systematic search of Medline, Embase and CINAHL online databases was undertaken for articles published in English between 1 January 2012 and 16 August 2024. A preliminary scoping review found the earliest eligible study on whole-food dietary interventions for paediatric CD was published in 2014, with increasing scientific attention from the mid-2010s. Consequently, we set the search period to begin in 2012. The search used a combination of subject headings, keywords and synonyms related to ‘paediatric CD’ and ‘dietary therapy’, as shown in Table S2. Full search description is provided in Table S3. The reference lists of potential eligible papers were manually screened (backward citation search) and citation lists of potential eligible papers in Web of Science were also scanned (forward citation search). Relevant reviews were manually screened for individual studies. Abstracts and study protocols led to searches for eligible full-text articles of the same studies.

### Eligibility criteria

#### Type of participant

Children (⩽18 years) diagnosed with CD. For studies with mixed populations (e.g. children and adults or paediatric IBD subtypes), stratified results for children with CD were required.

#### Types of interventions and comparisons

Whole-food diets designed for CD, either as monotherapy or in combination with partial enteral nutrition (PEN), were included. Comparators could include any alternative or variation, including regular diets. Studies without a comparator group were also considered.

#### Types of outcome measures

The primary outcome was clinical remission, defined by study-specific criteria using validated tools (e.g. PCDAI, Harvey Bradshaw Index, etc.). Remission rates were reported using intention-to-treat analysis for comparability. The secondary outcomes, when data were available, included dietary tolerance and adherence, mucosal healing, inflammatory biomarker changes, growth and nutrition and microbiota/metabolite shifts.

#### Types of studies

Randomised controlled trials (RCT), quasi-experimental studies, cohort studies, case-control and case series were included; however, case-series studies that solely reported individual outcomes were excluded. Both prospective and retrospective designs were considered. Detailed inclusion and exclusion criteria are summarised in Table S2.

### Study selection

Retrieved studies were imported into Covidence systematic review software (Veritas Health Innovation, Melbourne, VIC, Australia), and duplicate records were removed. Lead reviewer (C.G.) independently completed title and abstract screening. Two reviewers (C.G. and L.J.R.) independently conducted full-text screening. Results were compared, and any disagreements were resolved by a third reviewer (D.G.) through discussion.

### Assessment of evidence quality and risk of bias

The quality of evidence for included studies was evaluated using the National Health and Medical Research Council 6-point scale, with levels I (highest) to IV (lowest) representing levels of evidence.^15^ Studies were further assessed for their risk of bias using the ‘Quality Criteria Checklist – Primary Research’ tool created by the Academy of Nutrition and Dietetics Evidence Analysis Library,^16^ consisting of 10 validity appraisal criteria. Each study received a quality rating indicating its risk of bias. The three ratings were: ‘Positive’ for low risk of bias with issues clearly addressed, ‘Negative’ for high risk of bias with issues inadequately addressed and ‘Neutral’ for studies neither exceptionally strong nor exceptionally weak and with uncertain risk. The assessment for each study was performed independently by two reviewers (C.G. and L.J.R.). Results were compared and discussed with the research team to achieve an agreement on the final quality rating.

### Data extraction

An initial data extraction form was developed and refined by the research team until consensus was reached. Data were extracted independently by C.G. from eligible studies and checked by the research team. Data extracted included study characteristics, intervention characteristics for both intervention and comparator groups (where available) and intervention outcomes of interest.

### Data analysis

A meta-analysis of eligible studies was unfeasible due to the heterogeneity in intervention type, duration and outcomes. Instead, a critical synthesis was performed to provide an overview of the impact of whole-food diet therapies on remission and relevant health outcomes in children with CD across multiple studies.

## Results

**Figure 1.**
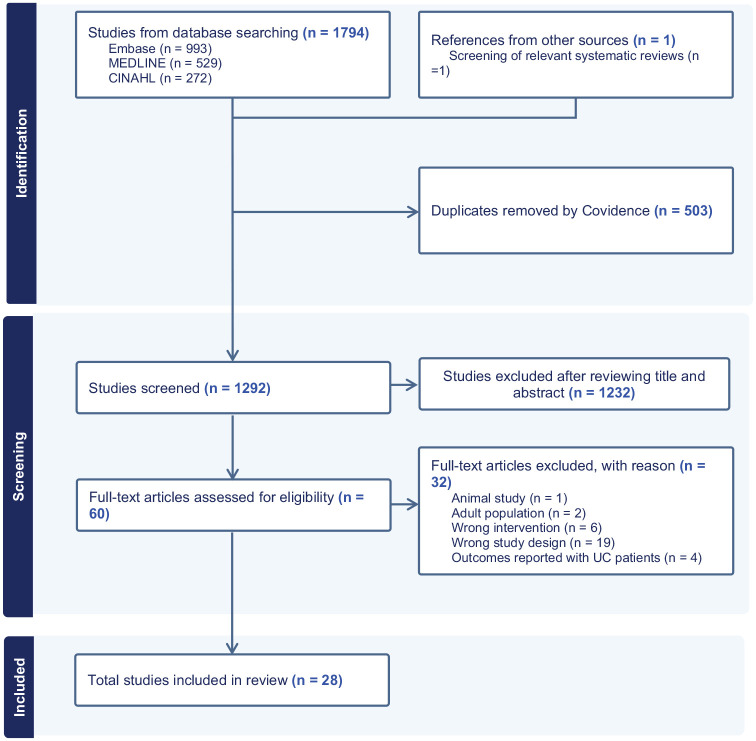
PRISMA flowchart of literature search and study selection. PRISMA, Preferred Reporting Item for Systematic Reviews and Meta-Analyses.

Figure 1 depicts the study selection process (PRISMA flowchart).^14^ A total of 1795 records were identified, and 60 studies proceeded to full-text review for eligibility. A total of 28 studies were subsequently included.

### Quality and risk of bias assessment

The 28 studies generally provided low- to medium-quality evidence, with more than half classified as III-3 level of evidence.^15^ For risk of bias, six studies received an overall ‘positive’ rating.^17–22^ One study received a ‘negative’ rating due to higher sources of bias,^23^ and the remaining 21 studies were rated as ‘neutral’, mostly due to unclear exclusion criteria, the absence of a comparator, incomparable groups, inadequate intervention descriptions and poor or unclear reporting blinding. Four post hoc analyses^24–27^ received a neutral rating because not all participants from the original trial were accounted for, and there was uncertainty about the reliability of using the original data to answer post hoc questions. The levels of evidence and quality assessment outcomes for the included studies are summarised in Table S4.

### Characteristics of the included studies

Table 1 outlines the characteristics of the 28 included studies. All studies were published within the past decade. These studies examined six unique whole-food therapies, including specific carbohydrate diet (SCD; *n* = 7, 25%),^18,23,28–32^ Crohn’s disease exclusion diet (CDED; *n* = 16, 57%),^17,19,20,24–27,33–41^ anti-inflammatory diet for Crohn’s disease (AID-CD; *n* = 2, 7%),^21,22^ plant-based diet (PBD; *n* = 1),^42^ Crohn’s disease treatment-with-eating diet (CD-TREAT; *n* = 1)^43^ and MD (*n* = 1).^44^ All SCD studies were conducted in the USA. Most CDED studies originated from Europe. Both AID-CD studies were conducted in Slovenia. Seven studies were controlled trials,^17,19,21,22,31,39,44^ 16 were observational studies (*n* = 2 with comparators)^18,23,28–30,32–38,40–43^ and 5 were post hoc analyses^20,24–27^ using subset data from one included RCT.^19^

**Table 1.** Characteristics of included studies (*n* = 28).

Author Year Country	Study design Sample size	Baseline participant characteristics^a^		Dietary intervention protocol for intervention (I) and comparator (C) groups	Intervention duration	Dietary adherence	Dietary tolerance
		Gender, age (mean ± SD/median (range) years), disease characteristic	Concomitant medication (%)				
SCD							
Suskind et al.^23^ 2014 USA	Retrospective single-arm cohort study *n* = 7	28% female Age: 11.3 ± 3.0 Mild to severe disease^b^ *n* = 3 (43%) treatment naïve	14% (1/7)	No details provided.	Mean 14.6 ± 10.8 (5–30) months	NR	NR
Cohen et al.^18^ 2014 USA	Prospective single-arm cohort study *n* = 10	30% female Age: 14 (10–17) Active, mild disease^c^ *n* = 3 (30%) newly diagnosed	60% (6/10)	Adapted from the Crohn’s and Colitis Foundation of America Website.	12 weeks extended to 52 weeks	3-day dietary records: Intake of 85% estimated energy requirement before diet vs 101% on the SCD. *n* = 1 not rigidly adherent to the diet.	90% (9/10)
Burgis et al.^28^ 2016 USA	Retrospective single-arm cohort study *n* = 11	27% female Age: 11.8 ± 3.0 *n* = 5 (45%) treatment naïve	73% (8/10)	Strict SCD for varying durations before adding additional foods based on personal preference.	52 weeks	Method is not reported. *n* = 3 on strict SCD *n* = 8 added extra foods at 7.7 ± 4.0 months.	NR
Obih et al.^29^ 2016 USA	Retrospective case-control study I: *n* = 20 C: *n* = 7	35% female Age: 10 (1.5–19) *n* = 13 active, moderate disease^d^ *n* = 14 in remission	45% (9/20)	I: No details provided. C: Patients began standard medical therapy as failed to maintain SCD >2 weeks.	Mean 9.6 ± 10.1 (3–48) months	Method is not reported. *n* = 10 on strict SCD (4–48 months). *n* = 10 added extra foods, e.g. rice, cheese, milk, potatoes.	74% (20/27)
Wahbeh et al.^32^ 2017 USA	Retrospective single-arm cohort study *n* = 7	57% female Age: 11.1 ± 3.4 Mild to severe disease^e^ *n* = 2 (29%) treatment naïve	0%	No details provided.	Median 26 (13–62) months	Method is not reported. No participant was on strict SCD who underwent follow-up endoscopic examination.	NR
Suskind et al.^30^ 2018 USA	Prospective single-arm cohort study, multicentre *n* = 9	Unable to identify^f^	Unable to identify^f^	A staged approach to introduce new foods toward the complete SCD.	12 weeks	3-day food intake record. Adherence NR	Unable to identify^f^
Suskind et al.^31^ 2020 USA	Double-blind RCT, single centre I: *n* = 5 C1: *n* = 5 C2: *n* = 4	43% female Age: 14.3 ± 2.9 Mild or moderate disease^g^	43% (6/14)	All participants were on SCD for the first 2 weeks before randomising onto their diet plan: I: SCD, no details. C1: Modified SCD (add oats, rice). C2: A whole-food-diet (eliminate wheat, corn, sugar, milk and food additives).	12 weeks	3-day food intake record. Adherence NR	I: 80% (4/5) C1: 80% (4/5) C2: 50% (2/4)
CDED							
Sigall Boneh et al.^34^ 2014 Israel	Retrospective single-arm cohort study *n* = 34	27% female Age: 13.2 ± 2.6 Most mild to moderate disease^h^ *n* = 10 (30%) newly diagnosed	62% (21/34)	Phase I CDED (1–6 weeks), phase II CDED (7–12 weeks) or CDED without PEN for 12 weeks.	12 weeks	Unable to identify^f^	NR
Sigall Boneh et al.^33^ 2017 Israel	Retrospective single-arm cohort study *n* = 10	30% female Age: 15.1 (12.7–17.5) *n* = 10 (100%) loss of response to biologics	100% (10/10)	Outpatients received phase I CDED (1–6 weeks), phase II CDED (7–12 weeks); hospitalised patients with severe relapses received EEN for 2 weeks before CDED.	12 weeks	Patient self-reporting: Compliance rate: 80% (8/10)	NR
Levine et al.^19^ 2019 Israel and Canada	RCT, multicentre I: *n* = 40 C: *n* = 38	I: 35% female Age: 13.8 ± 2.8 Mild to moderate disease^i^ C: 41% female Age: 14.5 ± 2.6 Mild to moderate disease^i^	I: 7.5% (3/40) C: 12% (4/34)	I: phase I CDED (1–6 weeks), phase II CDED (7–12 weeks). C: Standard care of EEN (1–6 weeks), free diet + 25% PEN (7–12 weeks).	12 weeks	Modiﬁed Medication Adherence Report Scale questionnaire; or direct questioning by dietitians using 72-h food diaries. compliance rate: I: 83% (33/40) C: 77% (26/34) (*p* = 0.52)	I: 98% (39/40) C: 74% (28/38) **(*p*** **=** **0.002)**
Sigall Boneh et al.^20^ 2021 Israel and Canada	Post hoc analysis I: *n* = 39 C: *n* = 34	37% female Age: 14.2 ± 2.7 Mild to moderate disease^i^	I: 7.7% (3/39) C: 12% (4/34)	Same as original trial.	Same as original trial.	Same as original trial	Same as original trial
Lev-Tzion et al.^26^ 2021 Israel and Canada	Post hoc analysis *n* = 45	39% female Age: 13.7 ± 2.9 Mild to moderate disease^i^	NR	Same as original trial.	Same as original trial.	Same as original trial.	Same as original trial.
Ghiboub et al.^25^ 2022 Israel and Canada	Post hoc analysis I: *n* = 22 C: *n* = 21	I: 36% female Age: 14.3 ± 2.8 Mild to moderate disease^i^ C: 33% female Age: 14.3 ± 2.5 Mild to moderate disease^i^	I: 14% (3/22) C: 19% (4/21)	Same as original trial.	Same as original trial.	Same as original trial.	Same as original trial.
Niseteo et al.^37^ 2022 Croatia	Retrospective case-control study I: *n* = 20 C: *n* = 41	I: 30% female Age: 13.9 (8.8–17.9) Mild to severe disease^j^ *n* = 18 (90%) newly diagnosed C: 59% female Age: 14.5 (6.7–17.9) Mild to severe disease^j^ All newly diagnosed	I: 75% (15/20) C: 85% (35/41)	I: phase I CDED, patients with severe disease received EEN for 1–2 weeks before CDED. C: standard care of EEN, oral intake or NG tube.	I: 6–8 weeks C: 6 weeks	NR	I: 90% (18/20) C: 93% (38/41)
Matuszczyk et al.^38^ 2022 Poland	Prospective single-arm cohort study *n* = 48	44% female Age: 13.4 (4–17) *n* = 23 (48%) newly diagnosed *n* = 17 (35%) in remission, the rest were with mild to moderate disease^k^	NR	Phase I CDED (1–6 weeks), phase II CDED (7–12 weeks).	12 weeks	Dietary consultations for assessing tolerance and adherence. *n* = 2 poor adherence	98% (47/48)
Stein et al.^39^ 2022 USA and Israel	Non-RCT, multicentre I: *n* = 11 C: *n* = 7	37% female Age: 16.2 ± 2.8 All in deep remission withdrawing from immunomodulator or anti-TNF monotherapy^l^	NR	Following medication withdrawal, participants chose to follow CDED or a free diet: I: CDED was extended to 52 weeks with two additional phases allowing limited free meals and/or home-cooked weekends. C: Participants who were unable to adhere to CDED >3 weeks were placed in free diet group.	52 weeks	Dietary recall and adherence questionnaires. Compliance rate: 67% (6/9)	NR
Ghiboub et al.^24^ 2023 Israel and Canada	Post hoc analysis I: *n* = 22 C: *n* = 21	I: 36% female Age: 14.3 ± 2.8 Mild to moderate disease^i^ C: 33% female Age: 14.3 ± 2.5 Mild to moderate disease^i^	I: 14% (3/22) C: 19% (4/21)	Same as original trial.	Same as original trial	Same as original trial	Same as original trial
Verburgt et al.^27^ 2023 Israel and Canada	Post hoc analysis I1: *n* = 32 I2: *n* = 22 C: *n* = 26 (healthy controls)	I1: 37% female Age: 14.3 ± 2.8 Mild to moderate disease^i^ I2: 32% female Age: 14.1 ± 2.1 Mild to moderate disease^i^ C: 46% female Age: 13.9 ± 6.2 years	I1: 9% (3/32) I2: 9% (2/22)	Same as original trial	Same as original trial	Same as original trial	Same as original trial
Arcucci et al.^17^ 2023 Argentina	RCT, multicentre I: *n* = 12 C: *n* = 10	I: 55% female Age: 10.6 (4.6–14.5) C: 20% female Age: 13.6 (12.7–14.4) All in apparent clinical remission^m^	I: 100% (11/11) C: 100% (10/10)	I: phase I CDED (1–6 weeks), phase II CDED (7–12 weeks). PEN was supplied via patients’ health insurance or by hospital. C: A healthy diet was advised with no specific food restrictions.	12 weeks	5-day dietary intake diary showed a good dietary compliance, several participants required the assistance of a dietitian to add variety to their diet.	92% (11/12)
Jijón Andrade et al.^35^ 2023 Spain	Retrospective single-arm cohort study *n* = 15	47% female Age: 13.9 (IQR 12–16) *n* = 9 (60%) treatment naïve *n* = 6 (40%) relapsed on biologics	100% (15/15)	Three phases of CDED according to the Modulife™ expert training programme.	24 weeks	Patients’ and parents’ impressions evaluated at follow-ups. Compliance rate: 100% (15/15)	NR
Martín-Masot et al.^36^ 2023 Spain	Prospective single-arm cohort study *n* = 24	38% female Age: 12.7 ± 2.9	NR	Three phases of CDED (no details provided).	52 weeks	Clinical interviews and 24-h dietary recalls demonstrated adequate adherence long term: protein intake from CDED required foods and a significant reduction in the consumption of foods excluded in CDED.	NR
Landorf et al.^40^ 2024 Australia	Retrospective single-arm cohort study n = 24	33% female Age: 13.8 ± 3.2 Mild to severe disease^h^ *n* = 20 (83%) treatment naïve	46% (11/24)	Phase I CDED (1–6 weeks), phase II CDED (7–12 weeks). Alternative PEN formulas offered to participants who were allergic to protein or vegan.	6 weeks with extension to 12 weeks	NR	NR
Scarallo et al.^41^ 2024 Italy	Retrospective single-arm cohort study n = 66	45% female Age: 12.3 (IQR 9.5–14.7) *n* = 19 (29%) severe disease^n^ *n* = 13 (22%) had either stricturing or penetrating *n* = 40 (61%) treatment naïve	39% (26/66)	Phase I CDED for 8 weeks (based on weight, not exceeding 1250 kcal/day).	8 weeks	A Likert scale ranging from 0 (lack of adherence) to 2 (complete adherence) Complete compliance rate: 82% (54/66)	NR
AID-CD (or modified CDED)							
Urlep et al.^21^ 2020 Slovenia	Non-RCT I: *n* = 12 C: *n* = 13	I: 46% female Age: 13.4 (9.8–17.9) *n* = 5 (36%) newly diagnosed C: 73% female Age: 13.8 (3.6–18.0) *n* = 9 (82%) newly diagnosed	I: 64% (7/11) C: 18% (2/11)	I: AID-CD for one meal per day (lunch or dinner) + 75% PEN. The AID-CD, adapted from the CDED, incorporates Slovenian cuisine and excludes fried foods. C: Standard care of EEN. Daily requirement based on age, gender, height, weight, physical activity plus an extra 20% of daily energy needs for catch-up growth.	6 weeks	NR	I: 92% (11/12) C: 85% (11/13)
Urlep et al.^22^ 2023 Slovenia	Non-RCT I: *n* = 14 C: *n* = 19	I: 38% female Age: 14.0 (IQR 4.5) *n* = 6 (46%) newly diagnosed C: 63% female Age: 14.1 (IQR 3.8) *n* = 12 (75%) newly diagnosed	I: 54% (7/13) C: 25% (4/16)	I: AID-CD for one meal per day (lunch or dinner) + 75% PEN. The AID-CD, adapted from the CDED, incorporates Slovenian cuisine and excludes fried foods. C: Standard care of EEN. Daily requirement based on age, gender, height, weight, physical activity plus an extra 20% of daily energy needs for catch-up growth.	6 weeks	Clinical interviews and 24-h dietary recalls questionnaire (amount and type of all foods and liquids, the volume of enteral formula consumed). Compliance rate: 100% for both groups	I: 93% (13/14) C: 84% (16/19)
PBD							
Chiba et al.^42^ 2017 Japan	Prospective single-arm cohort study *n* = 11	18% female Age: 15.9 ± 1.8 Moderate to severe disease^o^	100%	The lacto-ovo-semi-vegetarian diet included fish once a week and meat once every 2 weeks. Calories were gradually increased to a maximum of 30 kcal/kg standard body weight.	6 weeks	A significant high PBD score (mean 25) indicated a high adherence.	NR
CD-TREAT							
Svolos et al.^43^ 2019 UK	Prospective single-arm cohort study *n* = 5	20% female Age: 13.3 ± 3.2 Mild to moderate disease^p^ All had been treated preciously with EEN	80% (4/5)	The diet is prescribed and personalised, replicated nutritional profile of EEN (Modulen^®^) using whole food by excluding dietary components (i.e. gluten, lactose, alcohol) and closely matching macronutrients, vitamins, minerals and fibre (except for resistant starch).	8 weeks	Participants recorded daily dietary intake which demonstrated high compliance: actual energy intake 2298 kcal/day vs prescribed energy intake 2451 kcal/day	80% (4/5)
MD							
El Amrousy et al.^44^ 2022 Egypt	RCT I: *n* = 26 C: *n* = 28	Unable to identify^f^	NR	I: Received MD (KIDMED score ⩾8) C: Regular diet (KIDMED score ⩽7)	12 weeks	The KIDMED test Adherence NR	NR

a Baseline demographic is not statistically significant between intervention and control group unless indicated otherwise.

b Histologic severity based on the maximal amount of inflammation noted on biopsy.

c PCDAI = 15–30 mild, >30 moderate.

d PCDAI ⩾10 and ⩽30.

e SES-CD = 0 no ulcers, 1–2 inactive disease, 3–6 mild disease, 7–15 moderate disease, ⩾16 severe disease.

f Due to a mix reported with adult CD participants or paediatric ulcerative colitis participants.

g PCDAI >10 and 45.

h PCDAI = 7.5–27.5 mild, 30–37.5 moderate, >40 severe.

i PCDAI ⩾10 and ⩽40.

j Weighted PCDAI <12.5 remission, 12.5–40 mild, 41–57.5 moderate, >57.5 severe.

k PCDAI = 0–7.5 remission, 10–27 mild, 30–40 moderate, >40 severe.

l PCDAI <10, faecal calprotectin <250 µg/g, ileocolonoscopy results.

m PCDAI <10.

n Based on PGA.

o CDAI <150 quiescent stage, 150–220 mild–moderate, 220–450 moderate–severe, >450 severe/fulminant.

p Weighted PCDAI = 22.5–42.55.

AID-CD, anti-inflammatory diet for Crohn’s disease; CDED, Crohn’s disease exclusive diet; CD-TREAT, Crohn’s disease treatment-with-eating diet; EEN, exclusive enteral nutrition; IQR, interquartile range; KIDMED, Child and Adolescent Mediterranean Diet Quality Index; MD, Mediterranean diet; NG tube, nasogastric tube; NR, not reported; PBD, plant-based diet; PCDAI, paediatric Crohn’s disease activity index; PEN, partial enteral nutrition; PGA, Physician Global Assessment; RCT, randomised controlled trial; SCD, specific carbohydrate diet; SES-CD, Simple Endoscopic Examination for Crohn’s Disease.

The review included a total of 613 enrolled participants with CD (441 in intervention groups and 172 in comparator groups) with sample sizes ranging from 5 to 78 children. In most studies, more males than females participated with ages ranging from 1.5 to 19 years. One study that included a 19-year-old among its 20 participants was included in the review.^29^ While most studies included participants with active, mild to moderate CD, 4 studies had 71 participants in remission at baseline, accounting for 12% of the total participants.^17,29,38,39^ The median proportion of participants on concomitant medication was 60% (interquartile range (IQR) 39%, 85%). The concomitant medications included immunomodulators, biologics, immune suppressors and anti-inflammatory agents.

Dietary intervention protocols, including food selections, use of PEN, intervention durations and delivery methods, varied significantly across the six intervention diets. The median intervention durations of included studies was 12 weeks (IQR 8, 52 weeks), with only eight studies exceeding 24 weeks.^18,23,28,29,32,35,36,39^ Seventeen studies reported dietitian involvement.^17–19,21,22,30–38,40–42^ Most dietitians were specially trained in the intervention diets and provided dietary instruction, support and monitoring during fortnightly or monthly follow-ups.

Nineteen studies measured dietary adherence using 24-h dietary recall, food diaries, food frequency questionnaires or patient self-reports.^17–19,22,28–36,38,39,41–44^ Of the four SCD studies reporting dietary adherence, three indicated over half the participants consumed disallowed food items at various times.^18,28,29,32^ Studies that assessed dietary adherence of the other five diets found a median adherence rate of 83% (IQR 80%, 100%).

High tolerance rates (median 91%, IQR 80%, 94%) were identified from 10 studies.^17–19,21,22,29,31,37,38,43^ An RCT demonstrated CDED was significantly better tolerated compared to EEN (98% vs 74%, *p* = 0.002).^19^ The most common reasons for intolerance included having difficulty maintaining the diet, worsening symptoms and experiencing adverse events such as weight loss, nausea and vomiting.

### Overview of the included whole-food diet therapies

**Figure 2.**
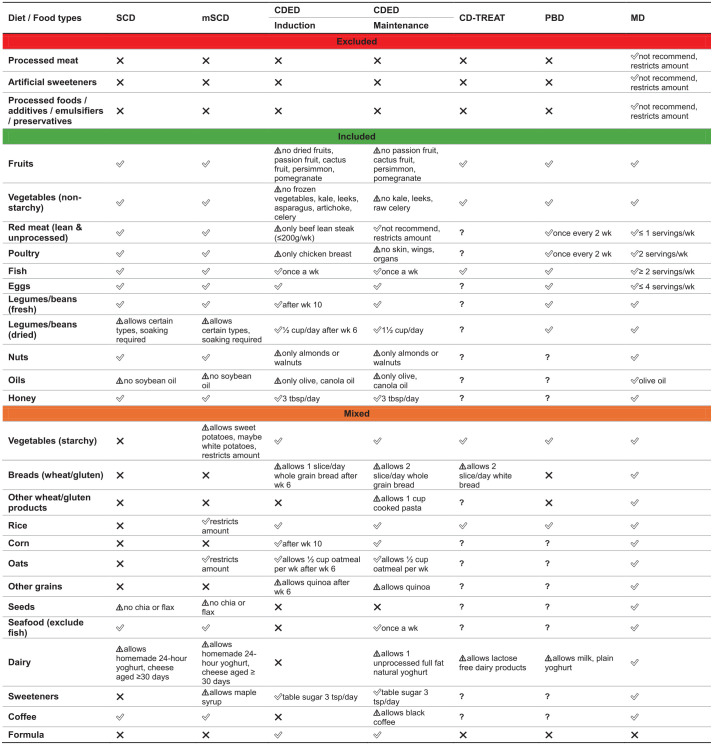
Comparison of key features across the included whole-food diet therapies. ✓, allowed; ⚠, partially allowed; **X**, restricted; **?**, unable to identify. CDED, Crohn’s disease exclusive diet; CD-TREAT, Crohn’s disease treatment-with-eating diet; MD, Mediterranean diet; mSCD, modified specific carbohydrate diet; PBD, plant-based diet; SCD, specific carbohydrate diet; wk, week.

Figure 2 provides a comparison of the core features of the whole-food diet therapies included in the review. While each diet varies in its structure, level of prescriptiveness and inclusion of PEN, several shared principles emerge: the emphasis on the reduction or exclusion of processed foods, food additives and refined sugars, alongside increased consumption of fruits, vegetables, legumes and healthy fats (e.g. from olive oil and fish). Most diets limit red meat and encourage lean protein sources, with some allowing whole grains, specific dairy products and different amounts of PEN depending on the treatment phase.

The SCD demonstrates a greater degree of restriction by excluding grains, cereals and certain starchy vegetables.^45^ The CDED is a phase-wised diet involving three phases: 2 × 6-week induction phases, and a third ‘maintenance’ phase. Phase I consists of 50% of energy requirements from a polymeric formula and 50% whole foods from a list of mandatory or allowed foods, while limiting insoluble fibre to prevent bowel obstructions. Phase II reduces the formula to 25% of requirements and reintroduces small portions of excluded foods such as vegetables. After remission is achieved, phase III introduces an increasing variety of foods to promote dietary flexibility and long-term compliance.^19^ The AID-CD is a culturally adapted modification of the CDED, incorporating elements of Slovenian cuisine while excluding fried foods.^21^ The CD-TREAT uses whole foods to mimic the nutritional profile of a polymeric formula used in EEN, matching its macronutrient, vitamin and mineral content, except for resistant starch, which is not present in EEN.^43^ The PBD is a lacto-ovo-semi-vegetarian diet that includes fish once per week and meat once every 2 weeks.^42^

### Clinical remission

Table 2 summarises the clinical remission rates in children with CD on whole-food diet therapies. Eighteen studies used various validated measures to assess clinical remission, with a median remission rate of 75% (IQR 62%, 85%).

**Table 2.** Clinical remission rates in children with Crohn’s disease on whole-food diet therapies (*n* = 18).

Study	Time	Remission rate^a^		*p*-Value
		Intervention group	Comparator group	
SCD				
Cohen et al.^18^	Week 12	60%	Nil	n.a.
	Week 52	86%		
Obih et al.^29^	Within 6 months	85%	Standard medications: 57%	n.s.
Suskind et al.^31^	Week 2	64%	Nil	n.a.
	Week 12	71%		
CDED				
Sigall-Boneh et al.^34^	Week 6	71%	Nil	n.a.
Sigall-Boneh et al.^33^	Week 6	60%	Nil	n.a.
Levine et al.^19^	Week 6	75%	EEN: 59%	*p* = 0.14
	Week 12	76%	Free diet + PEN: 45%	*p* = 0.01
Sigall Boneh et al.^20^	Week 3	62%	EEN: 65%	*p* = 0.78
Niseteo et al.^37^	Week 6–8	75%	EEN: 66%	*p* = 0.47
Matuszczyk et al.^38^	Week 12	55%	Nil	n.a.
Arcucci et al.^17^	Week 12	91%	Free diet: 60%	n.s.
Jijón Andrade et al.^35^	Week 6	100%	Nil	n.a.
	Week 24	77%		
Landorf et al.^40^	Week 6	71%	Nil	n.a.
	Week 12	53%		
Scarallo et al.^41^	Week 8	70%	Nil	n.a.
AID-CD				
Urlep et al.^21^	Week 6	75%	EEN: 69%	*p* = 0.99
Urlep et al.^22^	Week 6	78%	EEN: 68%	*p* = 0.69
PBD				
Chiba et al.^42^	Week 6	100%	Nil	n.a.
CD-TREAT				
Svolos et al.^43^	Week 8	60%	Nil	n.a.
MD				
El Amrousy et al.^44^	Week 12	92%	Free diet: 75%	NR

a Clinical remission is defined by study-specific criteria using validated measures, including PCDAI, Short PCDAI, Weighted PCDAI, Modified PCDAI, abbreviated PCDAI, CDAI, HBI, PGA. Significant difference between groups at *p* < 0.05. AID-CD, anti-inflammatory diet for Crohn’s disease; CDAI, Crohn’s Disease Activity Index; CDED, Crohn’s disease exclusive diet; CD-TREAT, Crohn’s disease treatment-with-eating diet; EEN, exclusive enteral nutrition; HBI, Harvey Bradshaw index; MD, Mediterranean diet; n.a., not applicable; NR, not reported; n.s., non-significant difference; PBD, plant-based diet; PCDAI, paediatric Crohn’s disease activity index; PEN, partial enteral nutrition; PGA, Physician’s Global Assessment; SCD, specific carbohydrate diet.

In the study by Obih et al.^29^ a higher proportion of participants achieved remission after an average of 9.6 months on the SCD compared to those who switched to medical therapy (85% vs 57%), no statistical difference was observed between the groups over time. In an RCT,^31^ 64% of participants achieved remission after 2 weeks on SCD before randomisation to either a modified SCD (adding oats, rice) or a whole-food diet (eliminating wheat, corn, sugar, milk and additives). The remission rate was sustained to week 12, indicating all three diets effectively improved PCDAI. However, statistical tests were not conducted due to small sample sizes.

Eleven CDED studies assessed remission in paediatric patients with diverse baseline disease characteristics, including newly diagnosed cases, relapse from previous treatments, loss of response to biologics and inactive disease. Three studies compared CDED to EEN for inducing CD remission, demonstrating that CDED (phase I) was as effective as EEN in inducing remission at 3 weeks (early remission) and 6 weeks.^19,20,37^ Of note, in the study by Niseteo et al.,^37^ 80% of participants in the CDED group received 1–2 weeks of EEN before starting CDED. Furthermore, three studies compared CDED to an unrestricted diet for maintaining CD remission. In the RCT by Levine et al.,^19^ the CDED group transitioned to the second phase of the diet after week 6, while the EEN group started a free diet with 25% PEN. At week 12, the CDED group had a significantly higher corticosteroid-free remission rate than the free diet with PEN group (76% vs 45%, *p* = 0.01). A subgroup analysis revealed a higher chance of maintaining remission in the CDED group compared to the free diet with PEN group at week 12 (87.5% vs 56%, *p* = 0.01). Another RCT by Arcucci et al.^17^ focused exclusively on children with CD in clinical remission at baseline (PCDAI < 10) and compared CDED to free diets for sustaining remission, showing that only one patient in the CDED group needed intensified biological treatment, compared to eight in the free diet group (*p* = 0.005) at week 12. Interestingly, one recent retrospective chart review observed participants with severe disease phenotype according to Physician’s Global Assessment were less likely to achieve clinical remission using CDED compared to those with mild to moderate disease (50% vs 80%, *p* = 0.012).^41^ Another retrospective study in Australia found CDED with formula modifications (using cow’s milk-based, rice-based or soy-based) appeared as effective as the original protocol.^40^ Even a small subset on CDED without formula saw remission in three of four children with CD.^34^

### Mucosal healing

Table 3 summarises mucosal healing criteria and rates reported in the included studies. Six studies assessed mucosal healing, five employing endoscopic procedures (reporting healing rates of 0%–60%),^18,21,22,32,42^ and one using the mucosal inflammation non-invasive (MINI) index (reporting a significant mucosal improvement).^36^ Among the five endoscopic studies, two (*n* = 1 SCD,^32^ *n* = 1 PBD^42^) used non-validated criteria, defining mucosal healing based on visible inflammation observed during endoscopy. Three studies used validated measures, including the Simple Endoscopic Examination for Crohn’s Disease score of 0 (*n* = 2 AID-CD) to quantify inflammation in the lower gastrointestinal tract,^21,22^ and a Lewis Score <135 (*n* = 1 SCD^18^) to assess small bowel inflammation via video capsule endoscopy.^46,47^

**Table 3.** Mucosal healing rates in children with Crohn’s disease on whole-food diet therapies (*n* = 6).

Diet study	Mucosal healing criteria	Time	Mucosal healing rate		*p*-Value
			Intervention group	Comparator group	
SCD Cohen et al.^18^	LS < 135 using capsule endoscopy.	Week 12	40%	Nil	n.a.
		Week 52	29%	Nil	
SCD Wahbeh et al.^32^	Absence of any ulceration from the upper gastrointestinal tract and ileocolon.	Median 26 (13–62) months	0%	Nil	n.a.
PBD Chiba et al.^42^	Absence of ulcer, aphthoid lesions, oedema, redness and bleeding by colonoscopy and/or contrast barium enema.	Week 6	60%	Nil	n.a.
AID-CD Urlep et al.^21^	Complete lack of endoscopically visible inflammation (SES-CD = 0).	Week 6	27%	EEN: 45%	*p* = 0.66
CDED Martín-Masot et al.^36^	MINI index <8 potentially indicates mucosal healing.	Week 52	Rate was not reported. However, mean MINI index decreased from 14.1 to 9.	Nil	*p* < 0.05
AID-CD Urlep et al.^22^	Complete lack of endoscopically visible inflammation (SES-CD = 0).	Week 6	38%	EEN: 44%	*p* = 0.99

Significant difference from baseline or between groups at *p* < 0.05.

AID-CD, anti-inflammatory diet for Crohn’s disease; CDED, Crohn’s disease exclusive diet; EEN, exclusive enteral nutrition; LS, Lewis Score; MINI, mucosal inflammation non-invasive; n.a., not applicable; PBD, plant-based diet; SCD, specific carbohydrate diet; SES-CD, simple endoscopic examination for Crohn’s disease.

The timing of endoscopic assessments varied across studies, ranging from 6 weeks to a median of 26 months. The highest mucosal healing rate (60%) was observed in the PBD study,^42^ which assessed healing at 6 weeks using non-validated measures to assess the lower gastrointestinal tract. Notably, all participants in this study received concomitant infliximab with the PBD intervention.^42^ In contrast, the lowest healing rate (0%) was reported in an SCD study with the longest intervention duration of 13–62 months (median 26).^32^ This study applied a stricter, non-validated definition of mucosal healing requiring the absence of ulceration in both the upper and lower gastrointestinal tracts. Despite the resolution of active CD symptoms and normalisation of inflammatory biomarkers, none of the participants achieved complete mucosal healing.^32^ Importantly, this SCD study used dietary intervention exclusively, without concomitant medications.^32^

### Laboratory inflammatory biomarkers

Table 4 provides an overview of the outcomes of laboratory inflammatory biomarkers reported in the included studies. Most participants on whole-food diet therapies achieved improvements in the inflammatory biomarkers, including FC, CRP, ESR and albumin.

**Table 4.** Outcomes of laboratory inflammatory biomarkers in children with Crohn’s disease on whole-food diet therapies (*n* = 19).

Study	Comparator group	Faecal calprotectin	C-reactive protein	Erythrocyte sedimentation rate	Albumin
SCD					
Suskind et al.^23^	Nil	↓^a^	Normalisation in *n* = 5/5	Normalisation in *n* = 2/4	Normalisation in *n* = 5/5
Cohen et al.^18^	Nil	NR	NR	n.s.	n.s.
Burgis et al.^28^	Nil	NR	NR	↓^a^	↑^a^
Obih et al.^29^	Standard medications	↓^a^ lower in SCD group^b^	↓^a^ lower in SCD group^b^	n.s.	n.s.
Wahbeh et al.^32^	Nil	n.s.	n.s.	NR	n.s.
Suskind et al.^31^	C1: mSCD C2: Whole food diet	NR	↓^c^ Normalisation in SCD and mSCD groups	↓^c^ Normalisation in SCD and mSCD groups	NR
CDED					
Levine et al.^19^	EEN + free diet	Week 6: ↓^a^ Week 12: n.s.	↓^a^	NR	NR
Sigall Boneh et al.^20^	EEN	NR	↓^a^	NR	NR
Niseteo et al.^37^	EEN	NR	↓^c^	NR	NR
Matuszczyk et al.^38^	Nil	↓^a^	↓^a^	↓^a^	NR
Arcucci et al.^17^	Free diet	↓^a^ in CDED group.	n.s.	n.s.	n.s.
Jijón Andrade et al.^35^	Nil	↓^a^ in treatment naïve subgroup.^d^ n.s. in relapsed on biologics subgroup.	n.s.	↓^a^ in treatment naïve subgroup.^d^ n.s. change in relapsed on biologics subgroup.	↑^a^ in treatment naïve subgroup.^d^ n.s. change in relapsed on biologics subgroup.
Martín-Masot et al.^36^	Nil	↓^a^	↓^a^	↓^a^	NR
Landorf et al.^40^	Nil	↓^a^	↓^a^	↓^a^	NR
Scarallo et al.^41^	Nil	↓^a^	↓^a^	↓^a^	↑^a^
AID-CD (or modified CDED)					
Urlep et al.^21^	EEN	↓^a^	↓^a^	↓^a^	↑^a^
Urlep et al.^22^	EEN	↓^a^	↓^a^	↓^a^	↑^a^
PBD					
Chiba et al.^42^	Nil	NR	↓^a^	NR	NR
CD-TREAT					
Svolos et al.^43^	Nil	↓^a^	NR	NR	NR

**↓**, Decrease from baseline; **↑**, increase from baseline.

a Significant difference from baseline in both groups (*p* < 0.05), unless otherwise specified.

b Significant difference between groups (*p* < 0.05) for changes.

c Significance not reported.

d Treatment naïve subgroup refers to newly diagnosed patients who have not received any prior treatment for CD, such as biological agents.

AID-CD, anti-inflammatory diet for Crohn’s disease; CDED, Crohn’s disease exclusive diet; CD-TREAT, Crohn’s disease treatment-with-eating diet; mSCD, modified specific carbohydrate diet; n.s., non-significant difference from baseline in both groups; NR, not reported; PBD, plant-based diet; SCD, specific carbohydrate diet.

Burgis et al.^28^ found that while participants on a strict SCD showed significant improvements in ESR and albumin, levels remained stable after the diet was liberalised. Two studies demonstrated that CDED was as effective as EEN in decreasing CRP levels.^19,37^ Chiba et al.^42^ reported that participants on a PBD plus Infliximab achieved a significant reduction in the mean CRP values, decreasing from 5.2 mg/dL at baseline to 0.2 mg/dL by week 6.

Twelve studies tracking FC showed reduced levels from baseline.^17,19,21–23,29,35,36,38,40,41,43^ Levine et al.^19^ found that CDED reduced FC levels comparably to EEN by week 6 (CDED: 3126–1744 µg/g; EEN: 2647–1021 µg/g). However, while the CDED group continued to show further reductions between weeks 7 and 12, FC levels in the EEN group rebounded to baseline after resuming a free diet. This finding aligned with those of Arcucci et al.,^17^ where participants on CDED experienced a significant FC reduction, while FC levels remained unchanged in those on free diets. Three CDED studies defined FC normalisation targets, achieving rates of 35% (<250 µg/g) by week 12,^38^ 27% (<150 µg/g) by week 8^41^ and 29% (<100 µg/g) by week 12.^40^

### Growth and nutritional parameters

Eleven studies reported increases in mean or median weight and body mass index (BMI) following whole-food dietary interventions.^17–19,21,23,28,29,37,38,40,41^ Levine et al.^19^ reported significant improvements in weight *z*-scores for both CDED and EEN by week 6 (*p* < 0.001), while Niseteo et al.^37^ found that CDED provided superior support for growth compared to EEN. In addition, Lev-Tzion et al.^26^ demonstrated significant improvements in bone formation following either CDED or EEN intervention. Martín-Masot et al.^36^ assessed children with CD adhering to CDED for up to 52 weeks, finding significant improvements in dietary habits and patterns.

### Faecal microbiota and metabolite

Two SCD^30,31^ and five CDED studies^19,24,25,27,39^ examined changes in faecal microbiota and/or metabolites associated with the dietary interventions. The SCD studies found highly individual changes in microbial composition but observed increased community diversity and improved dysbiosis after 12 weeks.^30,31^ The treatment was associated with an increase in *Blautia, Lachnospiraceae, Faecalibacterium, Roseburia, Anaerobutyricum* and *Eubacterium* and a decrease in *Escherichia coli* and *Faecalibacterium prausnitzii*.^31^ Suskind et al.^31^ also found that a strict SCD decreased the metabolism of starch and galactose while increasing the metabolism of plant polysaccharides, and caused a shift from amino acids biosynthesis to catabolism.

Levine et al.^19^ reported a decrease in Proteobacteria in participants from both CDED and EEN groups after 6 weeks. From weeks 7 to 12, the CDED group continued to show a decrease in Proteobacteria and an increase in microbial diversity. Conversely, the EEN group, following the reintroduction of free diets, had the microbial composition returning to baseline levels, notably with a major rebound in Proteobacteria. A subsequent post hoc analysis of metagenome sequences in faecal samples found that CDED with PEN increased Firmicutes, particularly *Clostridiales*, driven by genera including *Roseburia, Oscillibacter, Anaerotruncus* and *Ruminococcus*. It also decreased the relative abundance of Proteobacteria to the levels close to healthy controls, except for *E. coli*.^27^ Interestingly, Stein et al.^39^ found that the abundance of certain gut microbial gene pathways related to the metabolism of amino/nucleotide sugar and galactose from the baseline samples could predict a CD relapse at 52 weeks with 80% accuracy.

Ghiboub et al.^24,25^ observed a reduction in metabolites of the kynurenine pathway and an increase in those of the serotonin pathway in participants on CDED or EEN and in remission by week 12. However, in participants who failed to sustain remission, the metabolomic profile rebounded to baseline. Furthermore, Verburgt et al.^27^ found that although the concentration of short-chain fatty acid (SCFAs) was not associated with remission achieved by CDED with PEN by week 12, an increase in the SCFA synthesis pathway was observed.

## Discussion

This systematic review aimed to summarise the existing evidence regarding the impact of whole-food therapies on clinical remission and related health outcomes in children with CD. In total, 28 studies examined 6 unique types of diets that focus on modifying diet quality by incorporating nutrient-rich foods while excluding pro-inflammatory foods or components in children living with CD, demonstrating effects on clinical remission (*n* = 18/18 studies), mucosal improvement and healing (*n* = 5/6 studies), improved inflammatory biomarkers (*n* = 18/19 studies) and enhanced growth parameters (*n* = 11/13 studies). Outcomes related to gut microbiome changes, however, were inconsistent, and results should be interpreted with caution as most participants were on concomitant medication regimes and evidence overall was low to medium quality due to small, non-randomised, uncontrolled studies using varied definitions for clinical remission and mucosal healing outcomes. No conclusions could be made regarding the effects of whole-food diets on children with severe CD and complications, as most studies excluded this disease phenotype. Therefore, to overcome these methodological flaws more high-quality controlled intervention trials are needed to further support the efficacy of whole-food diet therapies in managing paediatric CD.

The included studies on whole-food diet therapies primarily focus on inducing clinical remission in children with uncomplicated, mild to moderate CD. A median remission rate of 75% (IQR 62%, 85%) was reported for these diets, which are comparable to the combined remission rates of 50%–90% observed for EEN in the literature.^48^ However, unlike EEN studies, most included studies on whole-food diets involved participants receiving additional medications. To minimise potential confounding effects from medications, most studies prohibited starting new medications or modifying existing doses during the dietary intervention or within a defined stabilisation period before initiating the diets. Participants, however, were typically allowed to continue stable doses of maintenance immunomodulators or biologics during the intervention. While this approach complicates the attribution of clinical remission solely to the diet, it reflects real-world clinical practice, where dietary interventions (except for EEN) are rarely used as standalone therapies for remission induction due to a lack of high-quality evidence. Further research is needed to investigate whole-food diets as adjuvant therapies alongside medications, including for severe CD. This approach may enhance real-world applicability and integration into routine CD management by leveraging the synergistic effects of medications targeting the immune response and dietary interventions addressing environmental triggers.^7^

In addition to combining with medications, most included studies, particularly those investigating the CDED and AID-CD protocols incorporated 25%–75% of total caloric intake from PEN. Although CDED with or without PEN demonstrated comparable efficacy in inducing remission in adults with mild to moderate biologic-naïve CD,^49^ its effectiveness in paediatric populations remains underexplored. The inclusion of PEN continues to be recommended in dietary protocols for children, as it helps meet daily energy and macro and micronutrient requirements (e.g. calcium) that may be difficult to achieve through CDED alone.^34^

Not surprisingly, remission rates for whole-food diet therapies varied significantly across studies, likely due to heterogeneity in study design, subjects enrolled, interventions and clinical remission criteria. Despite these variations, these diets share a focus on modifying diet quality by reducing processed foods high in fat, salt, refined sugar and additives that may exacerbate gut inflammation,^13^ while promoting nutrient-rich whole foods that positively influence the gut microbiota, metabolites and barrier function.^50^ While improving diet quality naturally minimises exposure to heavily processed foods, food additives and chemicals, the SCD is particularly restrictive by also excluding grains and starchy vegetables. This increased degree of restriction may contribute to its relatively lower adherence compared to other dietary therapies, as three studies reported over half of children consumed disallowed foods such as oats and potatoes during the intervention.^18,28,29^ However, adding these ‘illegal’ foods did not appear to compromise clinical remission rates or improvements in inflammatory biomarkers.^28,29^ These findings highlight the need for further research to understand the mechanisms driving gut inflammation and how these mechanisms may vary or align with individual biology, suggesting that some dietary restrictions may be unnecessary, and a more flexible diet tailored to the individual child might effectively achieve remission.

Among whole-food diet interventions, studies implementing CDED provided the most detailed dietary information and intervention protocols and have therefore been more widely replicated and investigated, contributing to a growing evidence base supporting its efficacy. Three studies comparing CDED to EEN showed similar remission rates at week 6,^19,20,37^ but CDED was better tolerated and more effective in sustaining remission by week 12.^19^ These outcomes align with recent guidelines, which suggest CDED be considered as an alternative to EEN to induce remission in paediatric patients with mild to moderate CD.^51^

The long-term efficacy of whole-food diet therapies in maintaining remission in children with CD remains unclear. Only four of the included studies explored clinical remission beyond 24 weeks, reporting a remission rate of 56%–86%.^18,29,35,39^ While these results appear promising, the clinical trials were statistically underpowered due to small sample sizes, highlighting a significant gap in understanding the effectiveness of whole-food diets in sustaining remission over time. Nevertheless, given the incomplete understanding of gut inflammation mechanisms, it is important to prioritise diet quality modifications over excessive dietary restrictions in long-term CD management. Diets rich in fruits, vegetables and fish have been linked to increased baseline microbial diversity, fewer disease flares and a delayed need for anti-TNF therapy in paediatric CD.^52^ These findings reinforce the value of a flexible, nutrient-balanced diet tailored to individual needs for long-term remission in paediatric CD.

Although dietary interventions show promise in achieving clinical remission in mild to moderate paediatric CD, their impact on mucosal healing remains underexplored. Mucosal healing, an objective treatment target in paediatric CD defined by the absence of ulcers on ileocolonoscopy, strongly predicts sustained clinical remission and better long-term outcomes compared to subjective clinical assessments.^6,46^ Among the studies reviewed, mucosal healing was only evaluated in six studies, consistently showing lower rates of healing compared to clinical remission.^18,21,22,32,36,42^ These findings align with the guidelines that highlight clinical scores alone do not adequately reflect mucosal healing.^8^ The six studies also demonstrated variability in the criteria used to define endoscopic healing, further complicating cross-study comparisons and the interpretation of results. Additionally, most studies had short follow-up durations (6 or 12 weeks), whereas guidelines recommend endoscopic outcome assessment at 6–9 months following the start of treatment to allow enough time for a treatment effect to be seen.^53^ However, this recommendation was tailored to patients treated with anti-TNF agents.^53^ In contrast, no trials to date have examined the optimal timing for evaluating mucosal healing in response to dietary interventions.

Although mucosal healing is an important long-term treatment target in paediatric CD,^6^ the invasive nature of endoscopy makes repeated assessments impractical and non-standard in most paediatric settings, posing a major barrier to routine monitoring of mucosal improvement. However, there is growing recognition that proxy measures such as the MINI index, FC and bowel ultrasound can serve as feasible alternatives to repeated ileocolonoscopies in children with CD.^54^ These non-invasive tools can complement established clinical indices, such as PCDAI, in evaluating treatment response and mucosal improvement.^54^ Future research should prioritise the development, validation and integration of non-invasive methods in dietary intervention studies to support the monitoring of mucosal healing over time, reduce procedural burden and enhance the feasibility of long-term disease monitoring in routine clinical practice.

Inflammatory biomarkers, particularly FC and CRP, are widely used to assess disease activity and response to therapy in CD.^55^ Most studies reviewed reported decreases in FC and CRP levels following dietary interventions, suggesting whole-food diet therapies may reduce intestinal and systemic inflammation. Current guidelines recommend reducing FC to <250 μg/g as a marker of treatment success,^8^ though a reliability study suggested that a combination of PCDAI < 10, CRP < 5 mg/dL and FC < 500 μg/g better predict inflammation resolution.^56^ However, variability in measurement criteria across included studies, including normalisation of values based on various thresholds, percentage reductions (e.g. >50% drop) or absolute reductions, introduces heterogeneity that complicates direct comparisons across studies.

Diet significantly influences gut microbiota composition and function, which are key regulators of metabolic and immune responses.^13^ Studies in this review found that, despite individual variation in faecal microbiome changes, remission achieved through SCD, CDED and EEN was associated with increased microbiome diversity and decreased abundance of Proteobacteria.^19,27,30,31^ At the genus level, successful SCD and CDED interventions promoted an increase in *Roseburia*,^27,31^ a key butyrate-producing bacterium in the colon that is typically depleted in patients with CD.^57^ Butyrate, an SCFA, is essential for maintaining intestinal barrier integrity and reducing inflammation.^58^ These results indicate that whole-food diet therapies may drive clinical improvement by restoring gut symbiosis through modulation of microbial composition and function. Notably, participants who relapsed following the reintroduction of regular diets or failed to respond to dietary therapies did not demonstrate those specific changes.^19,27^ This comparison raises questions about whether disease remission is driven by the modulation of the gut microbiome through dietary interventions or if remission itself leads to microbiome changes. Therefore, further exploration of the mechanistic links between gut microbiome and inflammation in CD is needed.

Adherence to therapeutic diets is often challenging and requires extensive support from family, friends and healthcare providers.^59^ A study assessing the feasibility of applying CDED reported 46% of participants had difficulty following the diet precisely, and 43% found it challenging to continue the diet beyond 12 weeks.^60^ However, with the exception of SCD, this review found relatively high adherence rates (median 83%, IQR 80%, 100%) across other dietary therapies, likely attributable to the predominantly short intervention durations (⩽12 weeks). It is also important to note that adherence measures in all included studies relied on subjective self-reports, which are prone to recall bias and under-reporting,^61^ highlighting the need for objective measures to assess dietary adherence. McKirdy et al.^62^ recently used faecal gluten immunogenic peptides (GIP) as an objective biomarker of compliance with EEN and found that 23% of participants deemed compliant by clinical review had detectable GIP, indicating gluten intake during EEN. These participants showed poorer responses to EEN compared to those with undetectable GIP. Since most whole-food diet therapies limit gluten, faecal GIP could potentially serve as a proxy biomarker for adherence to these diets.

Interestingly, even participants who struggled to maintain adherence to CDED beyond 12 weeks showed improvements in dietary habits, including increased fruit and vegetable intake and reduced consumption of processed foods,^63^ indicating the potential long-term benefits of these diets. Given the significant role of dietary adherence, dietary education and ongoing support from gastroenterology dietitians are essential for sustaining engagement with these therapies.^60^ To further enhance adherence and feasibility in clinical practice, future research should focus on developing clear dietary guidelines that address adherence barriers and enablers. Co-designing these guidelines with end users, including children with CD and their families, could ensure they are practical and adaptable to real-world clinical settings.

Whole-food diet therapies in paediatric CD requires careful consideration of nutritional and growth outcomes, as children with CD are particularly prone to malnutrition.^5^ While most studies included in this review assessed changes in growth parameters, such as weight, height and BMI, these measures alone were insufficient to provide a comprehensive evaluation of nutritional adequacy.^64^ Micronutrient deficiencies are significantly associated with CD and may persist despite improvements in growth parameters.^51^ For instance, a study evaluating SCD in a small cohort of children with IBD found that a significant percentage of children failed to meet daily requirements for calcium and vitamin D, despite achieving weight gain and adequate energy intake.^65^ Therefore, it is imperative that children undergoing therapeutic dietary interventions receive close monitoring and follow-up by dietitians (offered in some but not all studies), including anthropometric measurements and detailed evaluations of nutritional intake.

Beyond nutritional considerations, none of the included studies assessed the impact of whole-food dietary therapies on QoL in children and their families living with CD. As QoL is a key treatment goal,^6^ the evaluation of QoL is particularly relevant in studies comparing whole-food diets to EEN, where EEN is often associated with greater psychosocial burden due to the exclusion of solid foods. Future research should incorporate validated QoL measures to investigate how diet therapies affect overall well-being of the child and their family to inform the development of sustainable dietary strategies in paediatric CD.

**Figure 3.**
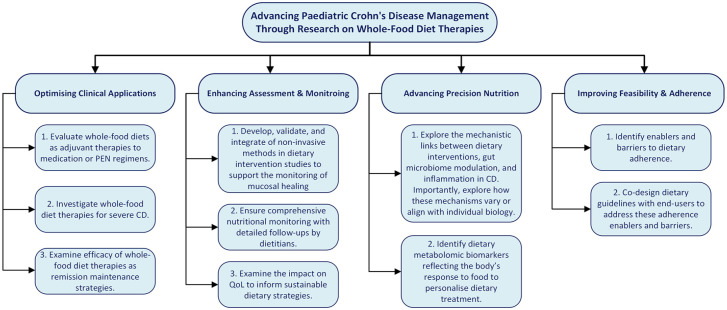
Future research directions for whole-food diet interventions in paediatric CD. CD, Crohn’s disease.

To the best of our knowledge, this is the first review to comprehensively evaluate all types of whole-food diet therapies studied specifically in the context of paediatric CD, whereas previous systematic reviews have exclusively focused on CDED in both children and adults with CD.^66,67^ This review highlights critical gaps in the current evidence base and identifies key priorities for advancing research on whole-food diet interventions in paediatric CD. Figure 3 presents a detailed summary of these recommendations, grouped into actionable categories.

Several limitations remain in our study. The diversity of whole-food diet therapies and heterogeneity in clinical remission criteria, study design and methodology hinder comparability and generalisation of findings; and the ability to perform a meta-analysis of dietary interventions across multiple studies with comparator groups. Additionally, clinical remission rates were often confounded by concomitant treatments, such as medications. Finally, the sample sizes in the included studies were generally small, leading to inadequate statistical power and a high risk of sampling and selection bias. Future research should recruit larger and diverse sample to ensure representativeness and adequate statistical power.

## Conclusion

In conclusion, our findings suggest that whole-food diet therapies hold potential for treating children with mild to moderate CD and that a flexible, nutrient-balanced dietary approach tailored to the individual child may be possible. However, the current evidence is limited by small, non-randomised and uncontrolled studies, which have employed inconsistent criteria to define clinical remission and involved varying types of concomitant medication regimes, preventing definitive conclusions. Compared to other whole-food diet therapies for paediatric CD, CDED has the stronger evidence base supporting its efficacy. However, studies examining its effectiveness in maintaining long-term remission remain limited. Therefore, large-scale, RCTs with standardised outcome measures and longer intervention durations are warranted to further support those results and to assess long-term efficacy. Furthermore, future research that explores the impacts of whole-food diets on the QoL and gut microbiome, identifies dietary metabolomic biomarkers for monitoring responses, and develops dietary guidelines that address adherence challenges will advance whole-food diets to precision nutritional therapies in paediatric CD.

## Supplemental Material

sj-docx-1-tag-10.1177_17562848251355436 – Supplemental material for Whole-food diet therapies for children with Crohn’s disease: a systematic review Supplemental material, sj-docx-1-tag-10.1177_17562848251355436 for Whole-food diet therapies for children with Crohn’s disease: a systematic review by Cathy Guo, Julia Fox, Kristie Bell, Danielle Gallegos and Lynda J. Ross in Therapeutic Advances in Gastroenterology

## Declarations

**Ethics approval and consent to participate:** Not applicable.

**Consent for publication:** All data utilised in this study were sourced from peer-reviewed journals, and no patient or participant consent was required.

**Availability of data and materials:** The data underlying this article are available in the article and in its online Supplemental Material.

## References

[1] Kuenzig ME Fung SG Marderfeld L , et al. Twenty-first century trends in the global epidemiology of pediatric-onset inflammatory bowel disease: systematic review. Gastroenterology 2022; 162(4): 1147–1159.e4. 10.1053/j.gastro.2021.12.282 34995526

[2] Ceballos D Hernández-Camba A Ramos L . Diet and microbiome in the beginning of the sequence of gut inflammation. World J Clin Cases 2021; 9(36): 11122–11147. 35071544 10.12998/wjcc.v9.i36.11122 PMC8717522

[3] Schirmer M Garner A Vlamakis H , et al. Microbial genes and pathways in inflammatory bowel disease. Nat Rev Microbiol 2019; 17(8): 497–511. 31249397 10.1038/s41579-019-0213-6 PMC6759048

[4] Grover Z Alex G . Management of inflammatory bowel disease in children: it is time for an individualised approach. J Paediatr Child Health 2020; 56(11): 1677–1684. 31613039 10.1111/jpc.14652

[5] Grover Z De Nardi A Lewindon P . Inflammatory bowel disease in adolescents. Aust Fam Physician 2017; 46: 565–571. 28787555

[6] Turner D Ricciuto A Lewis A , et al. STRIDE-II: an update on the selecting therapeutic targets in inflammatory bowel disease (STRIDE) initiative of the international organization for the study of IBD (IOIBD): determining therapeutic goals for treat-to-target strategies in IBD. Gastroenterology 2021; 160(5): 1570–1583. 33359090 10.1053/j.gastro.2020.12.031

[7] Gkikas K Svolos V White B , et al. An update on dietary therapies in paediatric Crohn’s disease. Curr Opin Clin Nutr Metab Care 2024; 27(3): 304–312. 38456807 10.1097/MCO.0000000000001024

[8] Van Rheenen PF Aloi M Assa A , et al. The medical management of paediatric Crohn’s disease: an ECCO-ESPGHAN guideline update. J Crohns Colitis 2020; 15(2): 171–194. 10.1093/ecco-jcc/jjaa161 33026087

[9] Forbes A Escher J Hébuterne X , et al. ESPEN guideline: clinical nutrition in inflammatory bowel disease. Clin Nutr 2017; 36(2): 321–347. 28131521 10.1016/j.clnu.2016.12.027

[10] Brown SC Wall CL Gearry RB , et al. Exclusive enteral nutrition for the treatment of pediatric Crohn’s disease: the patient perspective. Pediatr Gastroenterol Hepatol Nutr 2023; 26(3): 165–172. 37214167 10.5223/pghn.2023.26.3.165 PMC10192588

[11] Gatti S Vallorani M Quattrini S , et al. Dietary habits in Italian children with inflammatory bowel disease: a case-control multicenter study. J Pediatr Gastroenterol Nutr 2024; 79(3): 602–609. 39108157 10.1002/jpn3.12344

[12] Hartman C Marderfeld L Davidson K , et al. Food intake adequacy in children and adolescents with inflammatory bowel disease. J Pediatr Gastroenterol Nutr 2016; 63(4): 437–444. 26925608 10.1097/MPG.0000000000001170

[13] Levine A Sigall Boneh R Wine E . Evolving role of diet in the pathogenesis and treatment of inflammatory bowel diseases. Gut 2018; 67(9): 1726–1738. 29777041 10.1136/gutjnl-2017-315866

[14] Page MJ McKenzie JE Bossuyt PM , et al. The PRISMA 2020 statement: an updated guideline for reporting systematic reviews. BMJ 2021; 372: n71. 10.1136/bmj.n71 PMC8005924 33782057

[15] National Health and Medical Research Council. NHMRC additional levels of evidence and grades for recommendations for developers of guidelines: stage 2 consultation. Canberra: National Health and Medical Research Council, 2009.

[16] Academy of Nutrition and Dietetics. Quality criteria checklist – primary research 2016, https://www.andeal.org/evidence-analysis-manual (2016, accessed 10 October 2024).

[17] Arcucci MS Menendez L Orsi M , et al. Role of adjuvant Crohn’s disease exclusion diet plus enteral nutrition in asymptomatic pediatric Crohn’s disease having biochemical activity: a randomized, pilot study. Indian J Gastroenterol 2023; 43(1): 199–207. 37610564 10.1007/s12664-023-01416-x

[18] Cohen SA Gold BD Oliva S , et al. Clinical and mucosal improvement with specific carbohydrate diet in pediatric Crohn disease. J Pediatr Gastroenterol Nutr 2014; 59(4): 516–521. 24897165 10.1097/MPG.0000000000000449

[19] Levine A Wine E Assa A , et al. Crohn’s disease exclusion diet plus partial enteral nutrition induces sustained remission in a randomized controlled trial. Gastroenterology 2019; 157(2): 440. 31170412 10.1053/j.gastro.2019.04.021

[20] Sigall Boneh R Van Limbergen J Wine E , et al. Dietary therapies induce rapid response and remission in pediatric patients with active Crohn’s disease. Clin Gastroenterol Hepatol 2021; 19(4): 752–759. 32302709 10.1016/j.cgh.2020.04.006

[21] Urlep D Benedik E Brecelj J , et al. Partial enteral nutrition induces clinical and endoscopic remission in active pediatric Crohn’s disease: results of a prospective cohort study. Eur J Pediatr 2020; 179(3): 431–438. 31781933 10.1007/s00431-019-03520-7

[22] Urlep D Orel R Kunstek P , et al. Treatment of active Crohn’s disease in children using partial enteral nutrition combined with a modified Crohn’s disease exclusion diet: a pilot prospective cohort trial on clinical and endoscopic outcomes. Nutrients 2023; 15(21): 4676. 37960328 10.3390/nu15214676 PMC10650058

[23] Suskind DL Wahbeh G Gregory N , et al. Nutritional therapy in pediatric Crohn disease: the specific carbohydrate diet. J Pediatr Gastroenterol Nutr 2014; 58(1): 87–91. 24048168 10.1097/MPG.0000000000000103

[24] Ghiboub M Boneh RS Sovran B , et al. Sustained diet-induced remission in pediatric Crohn’s disease is associated with kynurenine and serotonin pathways. Inflamm Bowel Dis 2023; 29(5): 684–694. 36637175 10.1093/ibd/izac262 PMC10152286

[25] Ghiboub M Penny S Verburgt CM , et al. Metabolome changes with diet-induced remission in pediatric Crohn’s disease. Gastroenterology 2022; 163(4): 922. 35679949 10.1053/j.gastro.2022.05.050

[26] Lev-Tzion R Ben-Moshe T Abitbol G , et al. The effect of nutritional therapy on bone mineral density and bone metabolism in pediatric Crohn disease. J Pediatr Gastroenterol Nutr 2021; 72(6): 877–882. 33587407 10.1097/MPG.0000000000003073

[27] Verburgt CM Dunn KA Ghiboub M , et al. Successful dietary therapy in paediatric Crohn’s disease is associated with shifts in bacterial dysbiosis and inflammatory metabotype towards healthy controls. J Crohns Colitis 2023; 17(1): 61–72. 36106847 10.1093/ecco-jcc/jjac105 PMC9880954

[28] Burgis JC Nguyen K Park KT , et al. Response to strict and liberalized specific carbohydrate diet in pediatric Crohn’s disease. World J Gastroenterol 2016; 22(6): 2111–2117. 26877615 10.3748/wjg.v22.i6.2111 PMC4726683

[29] Obih C Wahbeh G Lee D , et al. Specific carbohydrate diet for pediatric inflammatory bowel disease in clinical practice within an academic IBD center. Nutrition 2016; 32(4): 418–425. 26655069 10.1016/j.nut.2015.08.025

[30] Suskind DL Cohen SA Brittnacher MJ , et al. Clinical and fecal microbial changes with diet therapy in active inflammatory bowel disease. J Clin Gastroenterol 2018; 52(2): 155–163. 28030510 10.1097/MCG.0000000000000772 PMC5484760

[31] Suskind DL Lee D Kim Y-M , et al. The specific carbohydrate diet and diet modification as induction therapy for pediatric Crohn’s disease: a randomized diet controlled trial. Nutrients 2020; 12(12): 3749. 33291229 10.3390/nu12123749 PMC7762109

[32] Wahbeh GT Ward BT Lee DY , et al. Lack of mucosal healing from modified specific carbohydrate diet in pediatric patients with Crohn disease. J Pediatr Gastroenterol Nutr 2017; 65(3): 289–292. 28825776 10.1097/MPG.0000000000001619

[33] Sigall Boneh R Sarbagili Shabat C Yanai H , et al. Dietary therapy with the Crohn’s disease exclusion diet is a successful strategy for induction of remission in children and adults failing biological therapy. J Crohns Colitis 2017; 11(10): 1205–1212. 28525622 10.1093/ecco-jcc/jjx071

[34] Sigall-Boneh R Pfeffer-Gik T Segal I , et al. Partial enteral nutrition with a Crohn’s disease exclusion diet is effective for induction of remission in children and young adults with Crohn’s disease. Inflamm Bowel Dis 2014; 20(8): 1353–1360. 24983973 10.1097/MIB.0000000000000110

[35] Jijón Andrade MC Pujol Muncunill G Lozano Ruf A , et al. Efficacy of Crohn’s disease exclusion diet in treatment-naïve children and children progressed on biological therapy: a retrospective chart review. BMC Gastroenterol 2023; 23(1): 225. 37386458 10.1186/s12876-023-02857-6 PMC10311743

[36] Martín-Masot R Herrador-López M Navas-López VM . Dietary habit modifications in paediatric patients after one year of treatment with the Crohn’s disease exclusion diet. Nutrients 2023; 15(3): 554. 36771261 10.3390/nu15030554 PMC9921286

[37] Niseteo T Sila S Trivić I , et al. Modified Crohn’s disease exclusion diet is equally effective as exclusive enteral nutrition: real-world data. Nutr Clin Pract 2022; 37(2): 435–441. 34339527 10.1002/ncp.10752

[38] Matuszczyk M Meglicka M Wiernicka A , et al. Effect of the Crohn’s disease exclusion diet (CDED) on the fecal calprotectin level in children with active Crohn’s disease. J Clin Med Res 2022; 11(14): 4146. 10.3390/jcm11144146 PMC9317017 35887910

[39] Stein R Daniel SG Baldassano RN , et al. Outcomes and predictors of sustained remission after drug withdrawal in pediatric Crohn disease. J Pediatr Gastroenterol Nutr 2022; 75(5): 608–615. 35976282 10.1097/MPG.0000000000003589

[40] Landorf E Hammond P Abu-Assi R , et al. Formula modifications to the Crohn’s disease exclusion diet do not impact therapy success in paediatric Crohn’s disease. J Pediatr Gastroenterol Nutr 2024; 78(6): 1279–1286. 38623960 10.1002/jpn3.12215

[41] Scarallo L Banci E De Blasi A , et al. A real-life pediatric experience of Crohn’s disease exclusion diet at disease onset and in refractory patients. J Pediatr Gastroenterol Nutr 2024; 79(3): 592–601. 38962891 10.1002/jpn3.12283

[42] Chiba M Tsuji T Nakane K , et al. Induction with infliximab and a plant-based diet as first-line (IPF) therapy for Crohn disease: a single-group trial. Perm J 2017; 21: 17–19. 10.7812/TPP/17-009 PMC5638637 29035182

[43] Svolos V Hansen R Nichols B , et al. Treatment of active Crohn’s disease with an ordinary food-based diet that replicates exclusive enteral nutrition. Gastroenterology 2019; 156(5): 1354–1367.e6. 10.1053/j.gastro.2018.12.002 30550821

[44] El Amrousy D Elashry H Salamah A , et al. Adherence to the Mediterranean diet improved clinical scores and inflammatory markers in children with active inflammatory bowel disease: a randomized trial. J Inflamm Res 2022; 15: 2075–2086. 35411169 10.2147/JIR.S349502 PMC8994055

[45] Haas SV Haas MP . The treatment of celiac disease with the specific carbohydrate diet; report on 191 additional cases. Am J Gastroenterol 1955; 23(4): 344–360. 14361377

[46] Sands BE Danese S Chapman JC , et al. Mucosal and transmural healing and long-term outcomes in Crohn’s disease. Inflamm Bowel Dis 2024; 30(10): 1767–1775. 39083264 10.1093/ibd/izae159 PMC11879194

[47] Klenske E Bojarski C Waldner M , et al. Targeting mucosal healing in Crohn’s disease: what the clinician needs to know. Therap Adv Gastroenterol 2019; 12: 1756284819856865. 10.1177/1756284819856865 PMC6572879 31236140

[48] Narula N Dhillon A Zhang D , et al. Enteral nutritional therapy for induction of remission in Crohn’s disease. Cochrane Database Syst Rev 2018; 4(4): CD000542. 10.1002/14651858.CD000542.pub3 PMC6494406 29607496

[49] Yanai H Levine A Hirsch A , et al. The Crohn’s disease exclusion diet for induction and maintenance of remission in adults with mild-to-moderate Crohn’s disease (CDED-AD): an open-label, pilot, randomised trial. Lancet Gastroenterol Hepatol 2022; 7(1): 49–59. 34739863 10.1016/S2468-1253(21)00299-5

[50] Zhang P . Influence of foods and nutrition on the gut microbiome and implications for intestinal health. Int J Mol Sci 2022; 23(17): 9588. 36076980 10.3390/ijms23179588 PMC9455721

[51] Bischoff SC Bager P Escher J , et al. ESPEN guideline on clinical nutrition in inflammatory bowel disease. Clin Nutr 2023; 42(3): 352–379. 36739756 10.1016/j.clnu.2022.12.004

[52] Dijk S Jarman M Zhang Z , et al. Pre-diagnosis diet predicts response to exclusive enteral nutrition and correlates with microbiome in pediatric Crohn disease. Nutrients 2024; 16(7): 1033. 38613066 10.3390/nu16071033 PMC11013084

[53] Peyrin-Biroulet L Sandborn W Sands BE , et al. Selecting therapeutic targets in inflammatory bowel disease (STRIDE): determining therapeutic goals for treat-to-target. Am J Gastroenterol 2015; 110(9): 1324–1338. 26303131 10.1038/ajg.2015.233

[54] Turner D Russell RK Wine E , et al. Response to FDA draft guidance on pediatric IBD drug approval trials: a consensus statement from the IBD Porto Group. J Pediatr Gastroenterol Nutr 2025; 80(1): 238–241. 39564649 10.1002/jpn3.12395

[55] Van Rheenen PF Aloi M Assa A , et al. The medical management of paediatric Crohn’s disease: an ECCO-ESPGHAN guideline update. J Crohns Colitis 2021; 15(2): 171–194. 10.1093/ecco-jcc/jjaa161 33026087

[56] Zubin G Peter L . Predicting endoscopic Crohn’s disease activity before and after induction therapy in children: a comprehensive assessment of PCDAI, CRP, and fecal calprotectin. Inflamm Bowel Dis 2015; 21(6): 1386–1391. 25851564 10.1097/MIB.0000000000000388 PMC4450968

[57] He P Yu L Tian F , et al. Dietary patterns and gut microbiota: the crucial actors in inflammatory bowel disease. Adv Nutr 2022; 13(5): 1628–1651. 35348593 10.1093/advances/nmac029 PMC9526834

[58] Nie K Ma K Luo W , et al. *Roseburia intestinalis*: a beneficial gut organism from the discoveries in genus and species. Front Cell Infect Microbiol 2021; 11: 757718. 34881193 10.3389/fcimb.2021.757718 PMC8647967

[59] Green N Miller T Suskind D , et al. A review of dietary therapy for IBD and a vision for the future. Nutrients 2019; 11(5): 947. 31035465 10.3390/nu11050947 PMC6566428

[60] Alsarhan A Aljasmi R Ajaka N , et al. Challenges in managing paediatric Crohn’s disease with Crohn’s disease exclusion diet (CDED): the first single-center study in the United Arab Emirates. Cureus 2023; 15(8): e43970. 10.7759/cureus.43970 PMC10515460 37746457

[61] Bailey RL . Overview of dietary assessment methods for measuring intakes of foods, beverages, and dietary supplements in research studies. Curr Opin Biotechnol 2021; 70: 91–96. 33714006 10.1016/j.copbio.2021.02.007 PMC8338737

[62] McKirdy S Russell RK Svolos V , et al. The impact of compliance during exclusive enteral nutrition on faecal calprotectin in children with Crohn disease. J Pediatr Gastroenterol Nutr 2022; 74(6): 801–804. 35192573 10.1097/MPG.0000000000003425

[63] Sigall Boneh R Westoby C Oseran I , et al. The Crohn’s disease exclusion diet: a comprehensive review of evidence, implementation strategies, practical guidance, and future directions. Inflamm Bowel Dis 2024; 30(10): 1888–1902. 37978895 10.1093/ibd/izad255 PMC11446999

[64] Casadei K Kiel J . Anthropometric measurement. StatPearls [Internet], StatPearls Publishing, 2022. 30726000

[65] Braly K Williamson N Shaffer ML , et al. Nutritional adequacy of the specific carbohydrate diet in pediatric inflammatory bowel disease. J Pediatr Gastroenterol Nutr 2017; 65(5): 533–538. 28825603 10.1097/MPG.0000000000001613 PMC5653423

[66] Zhu Z Lei Y Lin Z . Effects of Crohn’s disease exclusion diet on remission: a systematic review. Therap Adv Gastroenterol 2023; 16: 17562848231184056. 10.1177/17562848231184056 PMC10467299 37655057

[67] Correia I Oliveira PA Antunes ML , et al. Is there evidence of Crohn’s disease exclusion diet (CDED) in remission of active disease in children and adults? A systematic review. Nutrients 2024; 16(7): 987. 38613020 10.3390/nu16070987 PMC11013840

