# Supplementary material for: Whole-food diet therapies for children with Crohn’s disease: a systematic review
Source: Ther Adv Gastroenterol. 2025 Jul 23;18:17562848251355436. doi: 10.1177/17562848251355436 (PMC12290382; doi:10.1177/17562848251355436)
Supplement: sj-docx-1-tag-10.1177_17562848251355436 – Supplemental material for Whole-food diet therapies for children with Crohn’s disease: a systematic review [file sj-docx-1-tag-10.1177_17562848251355436.docx]

**Supplementary Materials**

**Supplement Table S1**. The Preferred Reporting Items for Systematic Reviews and Meta-Analyses (PRISMA) Checklist.

| **Section and Topic** | **Item #** | **Checklist item** | **Location where item is reported** |
| --- | --- | --- | --- |
| **TITLE** | | |  |
| Title | 1 | Identify the report as a systematic review. | P1 |
| **ABSTRACT** | | |  |
| Abstract | 2 | See the PRISMA 2020 for Abstracts checklist. | P2-3 |
| **INTRODUCTION** | | |  |
| Rationale | 3 | Describe the rationale for the review in the context of existing knowledge. | P4-5 |
| Objectives | 4 | Provide an explicit statement of the objective(s) or question(s) the review addresses. | P5 |
| **METHODS** | | |  |
| Eligibility criteria | 5 | Specify the inclusion and exclusion criteria for the review and how studies were grouped for the syntheses. | P6-7 & Supplement Table S2 |
| Information sources | 6 | Specify all databases, registers, websites, organisations, reference lists and other sources searched or consulted to identify studies. Specify the date when each source was last searched or consulted. | P6 & Supplement Table S3 |
| Search strategy | 7 | Present the full search strategies for all databases, registers and websites, including any filters and limits used. | Supplement Table S3 |
| Selection process | 8 | Specify the methods used to decide whether a study met the inclusion criteria of the review, including how many reviewers screened each record and each report retrieved, whether they worked independently, and if applicable, details of automation tools used in the process. | P7-8 |
| Data collection process | 9 | Specify the methods used to collect data from reports, including how many reviewers collected data from each report, whether they worked independently, any processes for obtaining or confirming data from study investigators, and if applicable, details of automation tools used in the process. | P7-8 |
| Data items | 10a | List and define all outcomes for which data were sought. Specify whether all results that were compatible with each outcome domain in each study were sought (e.g. for all measures, time points, analyses), and if not, the methods used to decide which results to collect. | P6-7 & Supplement Table S2 |
|  | 10b | List and define all other variables for which data were sought (e.g. participant and intervention characteristics, funding sources). Describe any assumptions made about any missing or unclear information. | P6-7 & Supplement Table S2 |
| Study risk of bias assessment | 11 | Specify the methods used to assess risk of bias in the included studies, including details of the tool(s) used, how many reviewers assessed each study and whether they worked independently, and if applicable, details of automation tools used in the process. | P7 |
| Effect measures | 12 | Specify for each outcome the effect measure(s) (e.g. risk ratio, mean difference) used in the synthesis or presentation of results. | N/A |
| Synthesis methods | 13a | Describe the processes used to decide which studies were eligible for each synthesis (e.g. tabulating the study intervention characteristics and comparing against the planned groups for each synthesis (item #5)). | N/A |
|  | 13b | Describe any methods required to prepare the data for presentation or synthesis, such as handling of missing summary statistics, or data conversions. | Supplement Table S2 |
|  | 13c | Describe any methods used to tabulate or visually display results of individual studies and syntheses. | N/A |
|  | 13d | Describe any methods used to synthesize results and provide a rationale for the choice(s). If meta-analysis was performed, describe the model(s), method(s) to identify the presence and extent of statistical heterogeneity, and software package(s) used. | P8 |
|  | 13e | Describe any methods used to explore possible causes of heterogeneity among study results (e.g. subgroup analysis, meta-regression). | N/A |
|  | 13f | Describe any sensitivity analyses conducted to assess robustness of the synthesized results. | N/A |
| Reporting bias assessment | 14 | Describe any methods used to assess risk of bias due to missing results in a synthesis (arising from reporting biases). | N/A |
| Certainty assessment | 15 | Describe any methods used to assess certainty (or confidence) in the body of evidence for an outcome. | N/A |
| **RESULTS** | | |  |
| Study selection | 16a | Describe the results of the search and selection process, from the number of records identified in the search to the number of studies included in the review, ideally using a flow diagram. | P9 & Figure 1 |
|  | 16b | Cite studies that might appear to meet the inclusion criteria, but which were excluded, and explain why they were excluded. | Figure 1 |
| Study characteristics | 17 | Cite each included study and present its characteristics. | P10-11 & Table 1 |
| Risk of bias in studies | 18 | Present assessments of risk of bias for each included study. | P10 & Supplement Table S4 |
| Results of individual studies | 19 | For all outcomes, present, for each study: (a) summary statistics for each group (where appropriate) and (b) an effect estimate and its precision (e.g. confidence/credible interval), ideally using structured tables or plots. | P18-27 & Figure 2 & Table 2-4 |
| Results of syntheses | 20a | For each synthesis, briefly summarise the characteristics and risk of bias among contributing studies. | N/A |
|  | 20b | Present results of all statistical syntheses conducted. If meta-analysis was done, present for each the summary estimate and its precision (e.g. confidence/credible interval) and measures of statistical heterogeneity. If comparing groups, describe the direction of the effect. | N/A |
|  | 20c | Present results of all investigations of possible causes of heterogeneity among study results. | N/A |
|  | 20d | Present results of all sensitivity analyses conducted to assess the robustness of the synthesized results. | N/A |
| Reporting biases | 21 | Present assessments of risk of bias due to missing results (arising from reporting biases) for each synthesis assessed. | N/A |
| Certainty of evidence | 22 | Present assessments of certainty (or confidence) in the body of evidence for each outcome assessed. | N/A |
| **DISCUSSION** | | |  |
| Discussion | 23a | Provide a general interpretation of the results in the context of other evidence. | P28-33 |
|  | 23b | Discuss any limitations of the evidence included in the review. | P28-33 |
|  | 23c | Discuss any limitations of the review processes used. | P33 |
|  | 23d | Discuss implications of the results for practice, policy, and future research. | P33 & Figure 3 |
| **OTHER INFORMATION** | | |  |
| Registration and protocol | 24a | Provide registration information for the review, including register name and registration number, or state that the review was not registered. | P3 & P6 |
|  | 24b | Indicate where the review protocol can be accessed, or state that a protocol was not prepared. | N/A |
|  | 24c | Describe and explain any amendments to information provided at registration or in the protocol. | N/A |
| Support | 25 | Describe sources of financial or non-financial support for the review, and the role of the funders or sponsors in the review. | N/A |
| Competing interests | 26 | Declare any competing interests of review authors. | P35 |
| Availability of data, code and other materials | 27 | Report which of the following are publicly available and where they can be found: template data collection forms; data extracted from included studies; data used for all analyses; analytic code; any other materials used in the review. | N/A |

*From:*  Page MJ, McKenzie JE, Bossuyt PM, Boutron I, Hoffmann TC, Mulrow CD, et al. The PRISMA 2020 statement: an updated guideline for reporting systematic reviews. BMJ 2021;372:n71. doi: 10.1136/bmj.n71

**Supplement Table S2**. Eligibility criteria and search strategy following the PICOS (Participants, Interventions, Comparisons, Outcomes, Study Design) framework for inclusion of studies.

| PICOS | Inclusion and Exclusion Criteria | Examples of Search Terms  (Key Words and Synonyms) | Examples of Subject Headings  (MEDLINE (MeSH2024)) |
| --- | --- | --- | --- |
| Participants | **Inclusion:** Children (≤ 18 years) diagnosed with CD using any established diagnostic method.  **Exclusion:** Children (≤ 18 years) with UC, Adult (≥ 18 years) with CD or UC, animal studies.^*^ | Paediatric, pediatric, child, children adolescents, young people, teenagers, youth | "Child+", "Child Health", "Infant+", "Child+", "Adolescent", "Pediatrics+", "Hospitals, Pediatric" |
|  |  | Crohn’s disease, Crohn | "Crohn Disease" |
| Intervention | **Inclusion**: All defined whole-food diets for treating CD, either as monotherapy or in combination with PEN.  **Exclusion**: Studies exclusively focused on EEN, PEN plus a regular diet, parenteral nutrition, nutrition supplementation. | Diet, dietary or nutritional therapy, dietary or nutritional intervention, feeding method or practice | "Nutrition Therapy+", MH "Diet+", "Diet Therapy+" |
| Comparisons | Any variation or alternative to the whole-food diet, including regular diet. Interventional studies without a comparator group were also included. | **-** | **-** |
| Outcomes (Primary) | Clinical remission (as defined by each study using validated measures using PCDAI, sPCDAI, wPCDAI, mPCDAI, abbreviated PCDAI, CDAI, HBI, PGA).^†^ | **-** | **-** |
| Outcomes (Secondary) | Dietary adherence and tolerance^‡^; mucosal improvement or healing, changes in laboratory inflammatory biomarkers (CRP, ESR, albumin, haemoglobin, haematocrit, and FC), changes in growth and nutritional parameters (BMI, weight, height, daily energy intake, and dietary habits and patterns), changes in faecal microbiota or metabolite. | **-** | **-** |
| Study Design | **Inclusion:** RCT, quasi experimental studies, cohort studies, case-control and case-series studies.  **Exclusion**: Case-series studies that reported interventional outcomes on individual basis, conference abstracts, commentaries, letters, individual case studies, systematic reviews, and meta-analyses. | **-** | **-** |

BMI = Body Mass Index; CD = Crohn’s Disease; CDAI = Crohn Disease Activity Index; CRP = C-Reactive Protein; EEN = Exclusive Enteral Nutrition; ESR = Erythrocyte Sedimentation Rate; FC = Faecal Calprotectin; HBI = Harvey Bradshaw Index; ITT = Intention-To-Treat; PCDAI = Paediatric Crohn Disease Activity Index; PEN = Partial Enteral Nutrition; PGA = Physician’s Global Assessment; RCT = Randomised Controlled Trials; SES-CD = Simple Endoscopic Examination for Crohn Disease; sPCDAI = Short Paediatric Crohn Disease Activity Index; UC = Ulcerative Colitis; wPCDAI = Weighted Paediatric Crohn Disease Activity Index.

^*^If stratified results for children with CD could not be identified, we contacted the authors to request a breakdown of results for children with CD. Studies were excluded if stratified results were not provided.

^†^The remission rate was derived directly from study data or, if not explicitly reported, calculated by dividing the number of participants achieving study defined remission, by the total number of participants (ITT analysis).

^‡^Dietary tolerance was reported as tolerance rates, which were either directly reported by the studies or determined based on dropouts due to diet intolerance, such as difficulty maintaining the diet or withdrawing from interventions due to adverse events.

**Supplement Table S3**. Detailed Search Strategy

| **MEDLINE Search** | |
| --- | --- |
| **#** | **Query** |
| S1 | MH "Crohn Disease" |
| S2 | TI (crohn's OR crohns OR crohn) |
| S3 | AB (crohn's OR crohns OR crohn) |
| S4 | S1 OR S2 OR S3 |
| S5 | MH "Nutrition Therapy+" |
| S6 | (MH "Diet+") OR (MH "Diet Therapy+") |
| S7 | TI (diet* OR (nutrition* N2 (therapy OR intervention OR treat*)) OR (feeding N2 (method OR intervention OR practice))) |
| S8 | AB (diet* OR (nutrition* N2 (therapy OR intervention OR treat*)) OR (feeding N2 (method OR intervention OR practice))) |
| S9 | S5 OR S6 OR S7 OR S8 |
| S10 | (MH "Child+") OR (MH "Child Health") |
| S11 | (MH "Infant+") OR (MH "Child+") OR (MH "Adolescent") |
| S12 | (MH "Pediatrics+") OR (MH "Hospitals, Pediatric") |
| S13 | TI (paediatric OR paediatrics) OR TI (pediatric OR pediatrics) OR TI child*OR TI (adolescent* OR "young people" OR teenagers OR youth) |
| S14 | AB (paediatric OR paediatrics) OR AB (pediatric OR pediatrics) OR AB child* OR AB (adolescent* OR "young people" OR teenagers OR youth) |
| S15 | S10 OR S11 OR S12 OR S13 ORS14 |
| S16 | S4 AND S9 AND S15 |
| **CINAHL Search** | |
| **#** | **Query** |
| S1 | MH "Crohn Disease" |
| S2 | TI (crohn's OR crohns OR crohn) |
| S3 | AB (crohn's OR crohns OR crohn) |
| S4 | S1 OR S2 OR S3 |
| S5 | (MH "Nutrition+") OR (MH "Infant Nutrition+") OR (MH "Child Nutrition") OR (MH "Adolescent Nutrition") |
| S6 | (MH "Diet Therapy+") OR (MH "Nutritional Support+") |
| S7 | (MH "Diet+") OR (MH "Restricted Diet+") |
| S8 | TI diet* OR TI (nutrition* N2 (therapy OR intervention OR treat*)) OR TI (feeding N2 (method OR intervention OR practice)) |
| S9 | AB diet* OR AB (nutrition* N2 (therapy OR intervention OR treat*)) OR AB (feeding N2 (method OR intervention OR practice)) |
| S10 | S5 OR S6 OR S7 OR S8 OR S9 |
| S11 | (MH "Child+") OR (MH "Child, Hospitalized") OR (MH "Child Health") |
| S12 | (MH "Adolescence+") OR (MH "Child+") |
| S13 | (MH "Hospitals, Pediatric") OR (MH "Pediatric Care+") |
| S14 | TI (paediatric OR paediatrics) OR TI (pediatric OR pediatrics) OR TI child*OR TI (adolescent* OR "young people" OR teenagers OR youth) |
| S15 | AB (paediatric OR paediatrics) OR AB (pediatric OR pediatrics) OR AB child* OR AB (adolescent* OR "young people" OR teenagers OR youth) |
| S16 | S11 OR S12 OR S13 OR S14 ORS15 |
| S17 | S4 AND S10 AND S16 |
| **Embase Search** | |
| **#** | **Query** |
| S1 | 'crohn disease'/exp |
| S2 | crohns:ab,ti OR crohn:ab,ti |
| S3 | 'crohn disease'/exp OR (crohns:ab,ti OR crohn:ab,ti) |
| S4 | 'nutrition'/exp |
| S5 | 'diet'/exp OR 'diet therapy'/exp |
| S6 | diet*:ti,ab |
| S7 | (nutrition NEAR/2 (therapy OR intervention OR treat*)):ti,ab |
| S8 | (feeding NEAR/2 (method OR intervention OR practice)):ti,ab |
| S9 | 'nutrition'/exp OR ('diet'/exp OR 'diet therapy'/exp) OR diet*:ti,ab OR (nutrition NEAR/2 (therapy OR intervention OR treat*)):ti,ab OR (feeding NEAR/2 (method OR intervention OR practice)):ti,ab |
| S10 | 'child'/exp OR 'age'/exp OR 'pediatrics'/exp |
| S11 | paediatric:ti,ab OR paediatrics:ti,ab OR pediatric:ti,ab OR pediatrics:ti,ab OR child*:ti,ab OR adolescent*:ti,ab OR 'young people':ti,ab OR teenagers:ti,ab OR youth:ti,ab |
| S12 | ('child'/exp OR 'age'/exp OR 'pediatrics'/exp) OR (paediatric:ti,ab OR paediatrics:ti,ab OR pediatric:ti,ab OR pediatrics:ti,ab OR child*:ti,ab OR adolescent*:ti,ab OR 'young people':ti,ab OR teenagers:ti,ab OR youth:ti,ab) |
| S13 | ('crohn disease'/exp OR (crohns:ab,ti OR crohn:ab,ti)) AND ('nutrition'/exp OR ('diet'/exp OR 'diet therapy'/exp) OR diet*:ti,ab OR (nutrition NEAR/2 (therapy OR intervention OR treat*)):ti,ab OR (feeding NEAR/2 (method OR intervention OR practice)):ti,ab) AND (('child'/exp OR 'age'/exp OR 'pediatrics'/exp) OR (paediatric:ti,ab OR paediatrics:ti,ab OR pediatric:ti,ab OR pediatrics:ti,ab OR child*:ti,ab OR adolescent*:ti,ab OR 'young people':ti,ab OR teenagers:ti,ab OR youth:ti,ab)) |
| S14 | ('crohn disease'/exp OR (crohns:ab,ti OR crohn:ab,ti)) AND ('nutrition'/exp OR ('diet'/exp OR 'diet therapy'/exp) OR diet*:ti,ab OR (nutrition NEAR/2 (therapy OR intervention OR treat*)):ti,ab OR (feeding NEAR/2 (method OR intervention OR practice)):ti,ab) AND (('child'/exp OR 'age'/exp OR 'pediatrics'/exp) OR (paediatric:ti,ab OR paediatrics:ti,ab OR pediatric:ti,ab OR pediatrics:ti,ab OR child*:ti,ab OR adolescent*:ti,ab OR 'young people':ti,ab OR teenagers:ti,ab OR youth:ti,ab)) AND [2012-2024]/py AND [english]/lim AND ([article]/lim OR [article in press]/lim OR [preprint]/lim) |

* Truncation wildcard, used at the end of the root of a search term to search for a concept with multiple spellings and various endings.

N2: Near operator, finds the words if they are a maximum of two words apart from one another, regardless of the order in which they appear.

**Supplement Table S4**. Levels of evidence and quality assessment ratings for included studies.

|  | Level of evidence^*^ | Clear research question | No selection bias | Comparable groups | Handling of withdrawals | Use of blinding | Intervention described | Outcomes & measurements valid | Statistical analysis appropriate | Conclusion supported & limitations discussed | funding or sponsorship bias unlikely | Overall |
| --- | --- | --- | --- | --- | --- | --- | --- | --- | --- | --- | --- | --- |
|  | **SCD** | | | | | | | | | | | |
| Suskind, 2014 | III-3 |  | + | -- | + |  | -- | -- |  | + | + | -- |
| Cohen, 2014 | III-3 | + | + | -- | + | + | + | + | + | + | + | + |
| Burgis, 2016 | III-3 | + |  | -- |  |  | + | + | + | + | + |  |
| Obih, 2016 | III-2 | + |  |  | + |  |  | + | + | + | + |  |
| Wahbeh, 2017 | III-3 | + |  | -- |  |  |  | + | + | + | + |  |
| Suskind, 2018 | III-3 | + |  | -- | + |  |  | + | + | + | + |  |
| Suskind, 2020 | II | + | + |  | + | + | + |  |  | + | + |  |
|  | **CDED** | | | | | | | | | | | |
| Sigall-Boneh, 2014 | III-3 | + | + | -- |  |  | + | + | + | + | + |  |
| Sigall-Boneh, 2017 | III-3 | + | + | -- | + |  | + | + | + | + |  |  |
| Levin, 2019 | II | + | + | + | + | + | + | + | + | + |  | + |
| Sigall-Boneh, 2021 | II | + | + | + | + | + | + | + |  | + |  | + |
| Lev-Tzion, 2021 | III-3 | + |  |  | + | + | + |  |  | + |  |  |
| Ghiboub, 2022 | III-2 | + |  | + | + | + | + |  |  | + |  |  |
| Niseteo, 2022 | III-2 | + |  |  | + |  |  | + | + | + |  |  |
| Matuszczyk, 2022 | III-3 | + | + | -- | + |  |  | + | + | + | + |  |
| Stein, 2022 | III-2 | + | + |  | + |  |  | + | -- | + |  |  |
| Ghiboub, 2023 | III-2 | + |  | + | + | + | + |  |  | + |  |  |
| Verburgt, 2023 | III-2 | + | + | + | + | + | + |  |  | + |  |  |
| Arcucci, 2023 | II | + | + | + | + |  | + | + |  | + | + | + |
| Jijón Andrade, 2023 | III-3 | + | + | -- | + |  | + |  | + | + |  |  |
| Martín-Masot, 2023 | III-3 | + | + | -- | + |  | -- |  | + | + |  |  |
| Landorf, 2024 | III-3 | + | + | -- | + |  |  | + | + | + | + |  |
| Scarallo, 2024 | III-3 | + | + | -- |  |  | + | + | + | + | + |  |
|  | **AID-CD** | | | | | | | | | | | |
| Urlep, 2020 | III-2 | + |  | + | + |  | + | + | + | + | + | + |
| Urlep, 2023 | III-2 | + |  | + | + |  | + | + | + | + | + | + |
|  | **PBD** | | | | | | | | | | | |
| Chiba, 2017 | III-3 | + |  | -- | + |  | + | + | + | + | + |  |
|  | **CD-TREAT** | | | | | | | | | | | |
| Svolos, 2019 | III-3 | + | -- | -- | + |  | + | + | + | + |  |  |
|  | **MD** | | | | | | | | | | | |
| Amrousy, 2022 | II | + | + | -- | + |  | -- | + | + | + | + |  |

^*^Level of evidence: levels I to IV represent the highest to lowest evidence (I = A systematic review of level II studies, II = A randomised controlled trial, III-1 = A pseudorandomised controlled trial, III-2 = A comparative study with concurrent controls, III-3 = A comparative study without concurrent controls, IV = Case studies.)

+ = positive, no source or low source of bias;  = neutral, median or unclear source of bias; -- = negative, higher source of bias.
